# Cellular senescence in cancer: clinical detection and prognostic implications

**DOI:** 10.1186/s13046-022-02555-3

**Published:** 2022-12-27

**Authors:** Andreas Domen, Christophe Deben, Jasper Verswyvel, Tal Flieswasser, Hans Prenen, Marc Peeters, Filip Lardon, An Wouters

**Affiliations:** 1 Center for Oncological Research (CORE), Integrated Personalized and Precision Oncology Network (IPPON), University of Antwerp, 2610 Wilrijk (Antwerp), Belgium; 2 Department of Oncology, Antwerp University Hospital (UZA), 2650 Edegem (Antwerp), Belgium

**Keywords:** Senescence, Oncogene-induced senescence, Therapy-induced senescence, SASP, Cancer, Detection, Prognosis

## Abstract

Cellular senescence is a state of stable cell-cycle arrest with secretory features in response to cellular stress. Historically, it has been considered as an endogenous evolutionary homeostatic mechanism to eliminate damaged cells, including damaged cells which are at risk of malignant transformation, thereby protecting against cancer. However, accumulation of senescent cells can cause long-term detrimental effects, mainly through the senescence-associated secretory phenotype, and paradoxically contribute to age-related diseases including cancer. Besides its role as tumor suppressor, cellular senescence is increasingly being recognized as an in vivo response in cancer patients to various anticancer therapies. Its role in cancer is ambiguous and even controversial, and senescence has recently been promoted as an emerging hallmark of cancer because of its hallmark-promoting capabilities. In addition, the prognostic implications of cellular senescence have been underappreciated due to the challenging detection and sparse *in* and ex vivo evidence of cellular senescence in cancer patients, which is only now catching up. In this review, we highlight the approaches and current challenges of *in* and ex vivo detection of cellular senescence in cancer patients, and we discuss the prognostic implications of cellular senescence based on *in* and ex vivo evidence in cancer patients.

## Background

### Cellular senescence in cancer

Cellular senescence is a cell state characterized by four interdependent hallmarks: (i) a durable and generally irreversible cell-cycle arrest; (ii) a senescence-associated secretory phenotype (SASP); (iii) macromolecular damage; and (iv) an altered metabolism [1]. Apart from the involvement in physiological processes, such as developmentally-programmed senescence [2, 3], tissue repair and wound healing [4, 5], cellular senescence is mainly a cellular stress response designed to eliminate damaged cells [6], and it is induced by numerous damage-inducing triggers, including ageing, DNA damage, reactive oxygen species, activation of oncogenes or inactivation of tumor-suppressor genes and inflammatory cytokines [1, 7].

Senescence was first described in vitro in human fetal diploid cell strains by Hayflick and Moorhead in 1961 to explain the finite lifespan of normal human cells as these do not proliferate indefinitely [8]. This phenomenon was already linked to cancer early on [9], as most cancer cells acquire the potential for unlimited cellular division and gain an infinite lifespan. During the following decades, the hypothesis that cellular senescence is an evolutionary homeostatic mechanism designed to irreversibly limit cell proliferation of damaged cells, which are at risk of malignant transformation, and to protect against cancer became more broadly accepted [10, 11]. However, the beneficial effect of cellular senescence in the context of (pre)malignant transformation rather results from the broader biological purpose of senescence, as an important mechanism, next to apoptosis, to eliminate many kinds of damaged cells in physiological and pathological processes, in order to maintain tissue homeostasis [6]. During normal embryogenic development, cellular senescence is a programmed mechanism that plays instructive roles [3], promotes tissue remodeling [2], and is also involved in tissue repair and wound healing [4, 5]. Cellular senescence is also considered as a crucial endogenous tumor suppressor mechanism. In this context, senescent cells have been identified in non-malignant and premalignant tissues in human tumor xenograft models such as lung adenomas [12], human benign melanocytic nevi [13], benign prostatic hyperplasia (BPH) [14], colon adenoma [15–17], precancerous urinary bladder [17] and intraepithelial prostatic neoplasia (PIN) [18] specimens. Oncogene-induced senescence (OIS) (i.e., senescence as a response to the activation of an oncogene or inactivation of a tumor-suppressor gene [19]) in transgenic mice has shown to suppress tumorigenesis of T cell lymphoma [20], prostate cancer [21], melanoma [22], lung adenocarcinoma [23] and pancreatic ductal adenocarcinoma [24]. This clearly marks the benefit of the senescence-associated growth arrest for preventing the expansion of pre- or fully malignant cells.

In fact, the idea that senescence only has a net positive effect on suppressing tumor growth was contradicted by the findings that senescent malignant [25] as well as non-malignant cells [26–29] are capable of driving tumor growth. Senescent cells stay metabolically active and can secrete a plethora of largely pro-inflammatory cytokines, chemokines, growth factors and matrix-remodeling proteases, collectively known as the SASP [30], capable of creating a protumorigenic microenvironment and driving tumorigenesis [31, 32]. Due to their genomic instability and the possibility to acquire additional mutations, cancer cells can also override the senescence-associated cell-cycle arrest and escape from the non-proliferative compartment [33–37]. Hence, the generally irreversible senescence-associated cell-cycle arrest is not necessary terminal for senescent cancer cells [1]. In addition, both non-malignant senescent cells and premalignant cells accumulate with ageing [38] due to an impaired clearing of senescent cells by the immune system over time [6] and accumulating oncogenic mutations acquired throughout life [39, 40], respectively. As such, the possibility of both occurring and interacting in close proximity increases in late life [38]. When this occurs, the SASP of non-malignant senescent cells can drive tumorigenesis of premalignant cells [38] opposing the net beneficial effect of senescent cells as a regulator of tissue homeostasis and tumor suppressor, paradoxically contributing to cancer development [38]. Besides its role as tumor suppressor, cellular senescence is increasingly being recognized as an in vivo response in cancer patients to various anticancer therapies (i.e., therapy-induced senescence (TIS) [41, 42]).

Taken together, the role of cellular senescence in cancer is ambiguous and even controversial, and senescence has recently been promoted as an emerging hallmark of cancer because of its hallmark-promoting capabilities [43]. In addition, the prognostic implications have been underappreciated due to the challenging detection and sparse in vivo and ex vivo evidence in cancer patients, which is only now catching up.

In this review, we first highlight the approaches and current challenges of *ex* and in vivo detection of cellular senescence in cancer patients. Next, we provide a comprehensive overview of available data regarding senescence in cancer patients, and discuss the prognostic implications of both OIS and TIS based on *ex* and in vivo evidence of cancer patients with solid tumors. Finally, we propose a simplified model for the observed differential prognostic outcomes of OIS and TIS in cancer patients.

## Main text

### Detection of cellular senescence in cancer patients

Identification and quantification of senescent cells in cancer patients in a clinical context is a challenging task since there are no specific and universal markers for senescent cells yet [1, 44]. Nonetheless, as an emerging hallmark of cancer [43], *ex* and in vivo evidence for cellular senescence residing in human tissue has gained more attention in the last decade [1] and efforts are made to accurately detect senescent cells in cancer patients. Below, we provide an overview of the different (pre)clinical approaches to detect cellular senescence in human tissue, pointing out the advantages and difficulties to implement these as clinical tools for the diagnosis and follow-up on cellular senescence in the context of a cancer patient.

#### *Ex vivo* detection in patient tissue samples

The best known and most widely used marker of cellular senescence is enhanced activity of acidic lysosomal β-galactosidase in senescent cells [45, 46], as lysosomes increase in number and size when cells become senescent [47]. The senescence-associated beta-galactosidase (SA-β-Gal) activity is often considered the gold standard for identifying senescent cells, despite SA-β-Gal activity was reported as a non-universal marker for cellular senescence [48]. Although absent in most proliferating and quiescent cells [44], SA-β-Gal activity is expressed in certain cell types (i.e., macrophages [49], bone marrow cells [50], melanocytes and sebaceous and eccrine gland cells [48]) and in vitro cells under certain cell culture conditions (i.e., confluence and serum starvation [51–53]) independent of a senescent cell state. Also, SA-β-Gal is not essential for senescence as cells can become senescent without expressing SA-β-Gal [54]. Of note, SA-β-Gal detection is only possible in fresh snap-frozen tissue samples [45], thus hampering its use in a clinical context.

To overcome the disadvantages of SA-β-Gal as marker for senescence, a biotin-linked Sudan Black B (SBB) analogue was designed to detect lipofuscin accumulation in senescent cells [45]. Lipofuscin is a non-degradable aggregate of oxidized lipids and proteins [55], that accumulate in lysosomes of senescent cells due to senescence-related lysosomal malfunction, and is considered a hallmark of cellular senescence [1, 56]. In contrast to the enzymatic SA-β-Gal activity, lipofuscin is preserved in fixed materials [51]. As such, detection of cellular senescence is feasible in formalin-fixed paraffin-embedded (FFPE) archival tissue samples using the SSB histochemical stain [45]. The interpretation of the assay requires some experience, as lipofuscin aggregates can be very small and background dirt can be wrongly interpreted as positive SBB-positive lipofuscin aggregates, comprising the overall sensitivity [45]. Interestingly, endogenous lipofuscin is linked to chronic liver disease and can be detected by autofluorescence in biopsied samples of human liver tissue [57]. As such, autofluorescence of lipofuscin in the context of cellular senescence could potentially be exploited to detect senescence in patient samples.

Other commonly used markers of cellular senescence are the cell cycle inhibitors p16^INK4a^ and p21^WAF1/Cip1^, as most senescence-inducing triggers lead to the activation of the cell cycle inhibitor pathways p53/p21^WAF1/Cip1^ and/or p16^INK4a^ [7] (Fig. 2). While p21^WAF1/Cip1^ expression occurs early after senescence induction and is reversible upon tumor suppressor protein p53 inactivation, p16^INK4A^ expression is frequently induced late after senescence induction and is irreversible upon p53 inactivation [44, 58, 59]. p21^WAF1/Cip1^ expression is therefore more likely to represent early cellular senescence, whereas p16^INK4a^ expression represents a more established and durable senescence response [44]. However, p21^WAF1/Cip1^ can be expressed by non-senescent cells in case of DNA-damage [60] and the genes encoding for p21^WAF1/Cip1^ and p16^INK4a^ (i.e., *CDKN1A* and *CDKN2A*, respectively) were not identified within the core transcriptome signature of senescent cells [61].

The senescence-associated cell cycle arrest, which occurs in G1 and possibly in G2 phase of the cell cycle [62], is marked by the absence of the proliferation marker Ki67 [1]. However, Ki67 is also absent in other cell states with a temporarily and durable cell-cycle withdrawal in G_0_, such as quiescence and terminally differentiated cells, respectively. 5-ethynyl-2′-deoxyuridine (EdU) is another proliferation markers and a thymidine analog that, when administered to cells, can incorporate into DNA during replication [44]. Unfortunately, EdU is not applicable for ex vivo tissue samples as active proliferation is required after biopsy.

**Fig. 1:**
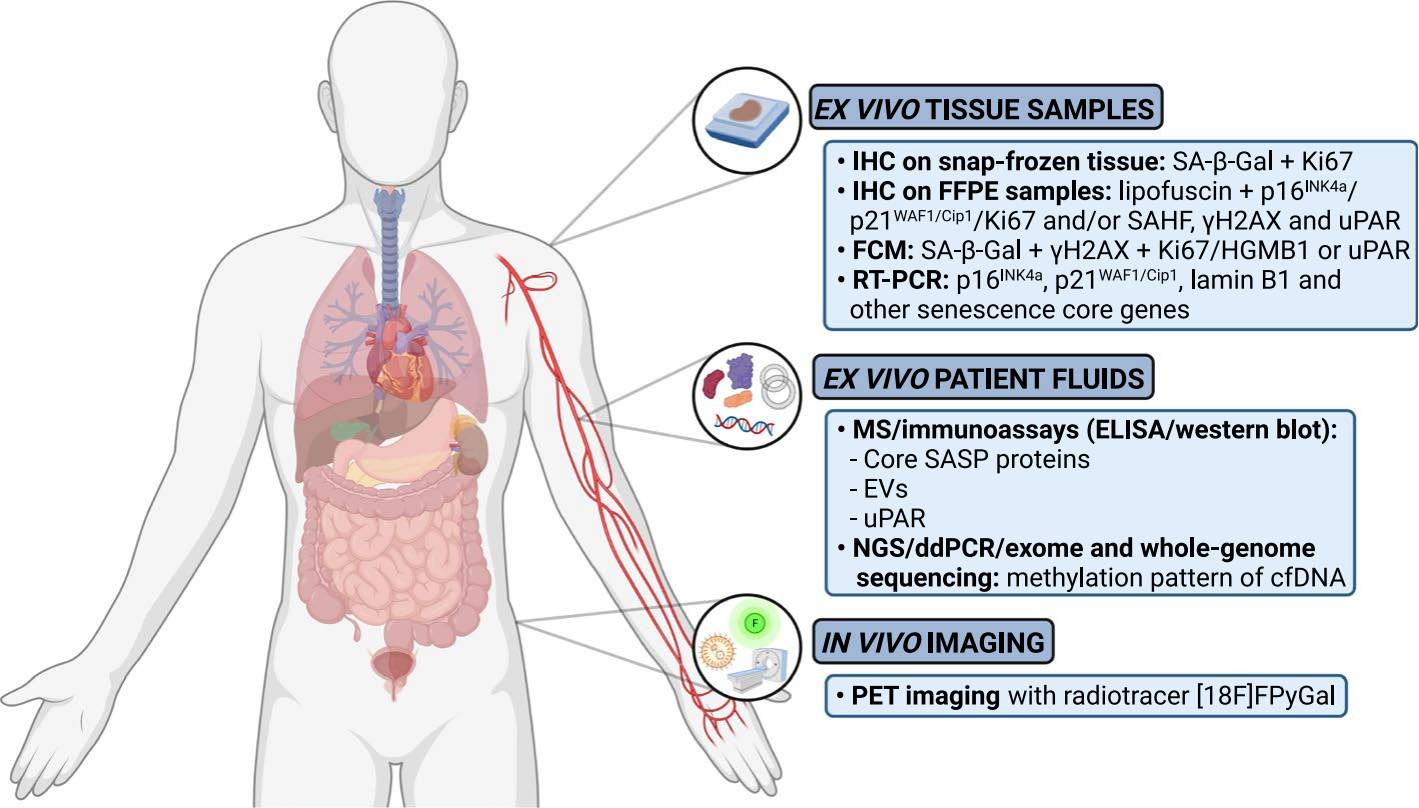
Overview of approaches of *ex* and in vivo detection of cellular senescence with corresponding senescence markers in cancer patients. IHC, immunohistochemistry; SA-β-Gal, senescence-associated beta-galactosidase; FFPE, formalin-fixed paraffin-embedded; SAHF, senescence-associated heterochromatin foci; γH2AX, phosphorylated H2AX; FCM, flow-cytometry; HGMB1, high mobility group box 1; RT-PCR; reverse transcription–polymerase chain reaction; MS, mass spectrometry; SASP, senescence-associated secretory phenotype; EVs, extracellular vesicles; NGS, next-generation sequencing; ddPCR, digital droplet polymerase chain reaction; cfDNA, cell-free DNA; PET-imaging, positron emission tomography-imaging

As SA-β-Gal activity [56], p16^INK4a^ and p21^WAF1/Cip1^ expression [60] and absence of Ki67 expression [63] are neither specific nor universal for cellular senescence, the International Cell Senescence Association [1] and others [44] recommend combining different markers for the detection of cellular senescence with the highest accuracy. However, combining all the markers in the same tissue sample is not yet possible without incurring artifactual false positives or negatives [44]. Therefore, current senescence validation in patient tissue samples can be determined either in snap-frozen samples using a sequential staining for SA-β-Gal and Ki67 on independent sequential and adjacent sections, or in FFPE tissue samples using a double-staining for lipofuscin and/or Ki67, p21^WAF1/Cip1^ or p16^INK4a^ combined with a sole staining of the remaining markers (Ki67, p21^WAF1/Cip1^ or p16^INK4a^) on sequential and adjacent Sects [44]. Additional immunohistochemical confirmation can be achieved by detection of components of senescence-associated heterochromatin foci (SAHF), including histone variant macroH2A [64], di- or trimethylated lysine 9 histone H3 (H3K9me2/3), heterochromatin protein (HP) 1 α, β and γ [64, 65] and high mobility group A (HMGA) proteins [64, 66, 67], and DNA damage foci such as phosphorylated H2AX (γH2AX) [68] (Fig. 1).

Using flow-cytometry, senescent cells can be identified and quantified on single-cell level by combining SA-β-Gal activity with staining of molecular markers for cellular senescence (e.g., γH2AX) and absence of Ki67 and/or high mobility group box 1 (HMGB1) protein [69]. Recently, a preliminary proof of concept method was developed to detect senescent cells with imaging flow cytometry based on measuring autofluorescence and morphological parameters, and on applying recent artificial-intelligence (AI) and machine learning (ML) tools [70], potentially facilitating cellular senescence detection without a multi-marker strategy.

Indirect markers of cellular senescence such as messenger RNA (mRNA) expression of p16^INK4a^, p21^WAF1/Cip1^ and lamin B1 (i.e., a nuclear lamina component and downregulated in case of senescence [71]), and selected senescence core genes can be determined by reverse transcription–polymerase chain reaction (RT-PCR) [44] (Fig. 1). However, both flow-cytometry and RT-PCR techniques require tissue dissociation and case-specific control samples, and do not provide any spatial information on senescent cells residing in the tissue, limiting their clinical utility for solid tumors. Although various senescence core genes have been determined [72], the senescent phenotype is dynamic and heterogeneous and depends on the tissue of origin and senescence-inducing trigger [72–77]. It is currently unclear which specific core genes should be included in order to confirm cellular senescence in a disease- and tissue-specific context [44]. Recently, using machine learning, a gene expression classifier (SENCAN classifier) was developed for the detection of senescence in cancer samples. Using transcriptome data as input, the SENCAN classifier was able to classify whether cancer cells are senescent or not. Unfortunately, whereas the SENCAN classifier is able to accurately detect senescence in many cancer cells in vitro, its accuracy to detect senescence in in vivo cancer samples is still unclear [76].

Of note, uPAR was recently identified as a cell surface protein that is broadly and specifically upregulated in senescent cells of mice using RNA-sequencing datasets derived from three independent and robust mouse models of OIS as well as TIS [78]. uPAR is involved in many intracellular signaling pathways that promote cell motility, invasion, proliferation and survival [79] and is expressed by tumor and stromal cells in a wide variety of human cancers where its expression frequently indicates poor prognosis [79]. In this context, uPAR expression and secretion (i.e., soluable uPAR) could be an interesting biomarker of senescence in cancer patients detected by immunohistochemistry, flow-cytometry or enzyme-linked immunosorbent assay (ELISA) [78] (Fig. 1).

#### *Ex vivo* detection in patient fluids

Detection of tumor-specific circulating material in patient fluids by means of liquid biopsy is an emerging field in oncology, with important clinical implications for personalized medicine [80]. In contrast to surgical or biopsy tissue samples, liquid biopsies are not subject to sampling bias, tumor heterogeneity and can be obtained repeatedly to monitor the evolution of the molecular profile of the tumor which may cause drug resistance [81]. However, the detection of cellular senescence via liquid biopsy in patient blood or urine requires specific senescence-associated circulating material including proteomes, extracellular vesicles (EVs) and circulating cell-free DNA (cfDNA).

First, SASP proteins can be measured by mass spectrometry or immunoassays (ELISA, western blot) in patient plasma [44, 74]. A proteomic atlas of core SASP secreted proteins originating from multiple senescence inducers and cell types was recently determined [74], enabling senescence detection by the presence of core SASP proteins, such as growth/differentiation factor 15 (GDF15), matrix metalloproteinase-1 (MMP1), stanniocalcin-1 (STC1), tissue inhibitor of metalloproteinases 1 and 2 (TIMP1 and TIMP2) [44] (Fig. 1). However, several of these core soluble SASP proteins have also been identified as biomarkers of human disease [75] and are positively associated with age, frailty and adverse post-surgery outcomes [82]. For example, GDF15, MMP1 and STC1 have been identified as a biomarker for cardiovascular disease [83], several cancers [84] and for Alzheimer’s disease [85], respectively. SASP biomarkers currently lack sensitivity to detect and attribute senescence in patient plasma to specific pathologies, including cancer [75]. However, SASP protein profiles differ among cell type, senescence-inducing trigger and interval after senescence induction [62, 74, 75], as well as age category [82] as senescent cells accumulate with increasing age [6]. By determining disease-, tissue-, and inducer-specific SASP factors as well as robust core SASP factors secreted by senescent cells in multiple contexts [75], it will become possible to attribute the secretion of certain SASP factors to the presence of senescent cells in patients in the near future.

Next to SASP proteins, senescent cells of human origin (i.e., foreskin primary [86], normal lung [87] and diploid [88] fibroblasts, prostate [87, 89], hepatocellular [87] and triple negative breast [90] cancer cells, retinal pigment epithelial cells [88] and human chondrocytes [91]) are capable of releasing EVs in patient fluids such as blood and urine [92]. (Fig. 1). EVs are small, lipid-bilayer enclosed, cell-derived particles that bear surface molecules that allow them to target recipient cells and contain transmembrane and enclosing cytosolic proteins and RNA [75, 93]. Once internalized, EVs release their content into the cytosol modifying the physiological state of the recipient cell [93] and enabling cell communication with neighboring as well as distant cells. As for SASP proteins, EV production and content drastically differ in physiological [94] and pathological [92] conditions, making EVs an additional interesting source of disease biomarkers. EVs of senescent cells are capable of transmitting paracrine senescence to neighboring cells [86, 91], contain chemotherapy and key proteins involved in cell proliferation after chemotherapeutic challenge [90] and can even promote cancer cell proliferation [88]. Interestingly, protein content of EVs secreted by senescent cells differs from secreted SASP proteins [74, 86], suggesting that SASP and EVs do not act as surrogate biomarkers and have different clinical significance and value [75]. However, as for SASP proteins, it is still unclear how EVs secreted by senescent cells exactly behave in physiologic and disease-specific contexts, and to what extend they depend on age, tissue and senescence-inducing trigger [75].

Detection and analysis of cfDNA by means of next-generation sequencing, digital droplet polymerase chain reaction, exome or whole-genome sequencing [95] could be a third appealing strategy to detect and monitor the senescence burden in cancer patients. Senescent cells exhibit a DNA methylation pattern of promoter hypermethylation mainly involving metabolic regulators, whereas transformed cells exhibit a DNA methylation pattern of promoter hypermethylation involving primarily pro-survival and developmental genes [96] (Fig. 1). Also, using a machine learning based approach trained with different early passage and senescent cells, a DNA methylation fingerprint of cellular senescence (DNAmSen) was developed and validated in clinical patient samples, such as whole blood and skin tissue [97]. With this approach, clear and robust correlations were found between the patient’s age and DNAmSen present in the corresponding sample. Interestingly, also elevated DNAmSen were observed in lung samples from patients with COPD and lung cancer compared to those of healthy controls [97]. Not surprisingly, the release of cfDNA is affected by type of treatment and timing from treatment exposure, and also heavily depend on the cellular response to treatment. Of note, it is thought that blocks the release of cfDNA whereas apoptosis and necrosis are key contributor of its release [98].

#### *In vivo* detection in patients

Currently, there is no established method to detect cellular senescence in vivo in patients. Detection can be achieved by chromogenic [99, 100] or fluorogenic [101–107] probes, preferentially hydrolyzed by SA-β-Gal, resulting in color- or fluorescence-enhanced senescent cells. Chromogenic [108] or fluorogenic [109] probes hydrolyzed by other lysosomal hydrolases overexpressed in senescent cells, such as α-L-fucosidase [110], can be used as well. Nanoparticles containing fluorescent dyes and probes have been developed that selectively release their content when the cap of the nanoparticle is hydrolyzed by SA-β-Gal after endocytosis [111–113], or by interaction with CD9 receptors [114] or β2 microglobulin [115], both preferentially expressed by senescent cells. Due to low tissue penetrance and autofluorescence the clinical use of these fluorescent probes and nanoparticles may be limited in patients [116]. An alternative method could be the detection of endogenous lipofuscin as endogenous lipofuscin, next to ex vivo in patient samples, can also be monitored in vivo and non-invasively via imaging, as has been shown in mice with chronic liver disease [57].

It should be noted that all these approaches were validated only in vitro or in vivo in mice and its use in patients should be further investigated. Currently, there is one first-in-human trial in cancer patients evaluating the safety and imaging characteristics of a novel senescence-specific radiotracer [18F]FPyGal (i.e., a radioactive form of SA-β-Gal) that can be tracked non-invasively in the body through positron emission tomography (PET) imaging (SenPET; NCT04536454) [117] (Fig. 1). Whether this strategy is sufficient to detect all senescent cells [118] due to the aforementioned limitations of SA-β-Gal as a specific marker of senescence has yet to be determined.

### Prognostic implications of cellular senescence in cancer patients

Senescence is considered to exert beneficial effects by halting cancer development and promoting survival in early life, but it is proposed to have detrimental effects later in life when senescent cells accumulate due to ageing and/or inappropriate removal [32, 38, 119]. Based on preclinical cancer research, these antagonistically pleiotropic effects of senescence are thought to be highly dependent on the type of cancer and senescence trigger [30, 32, 62]. The prognostic implications of cellular senescence in cancer are therefore often unpredictable primarily due to the dual role of the SASP [32].

#### Senescence burden

**Fig. 2:**
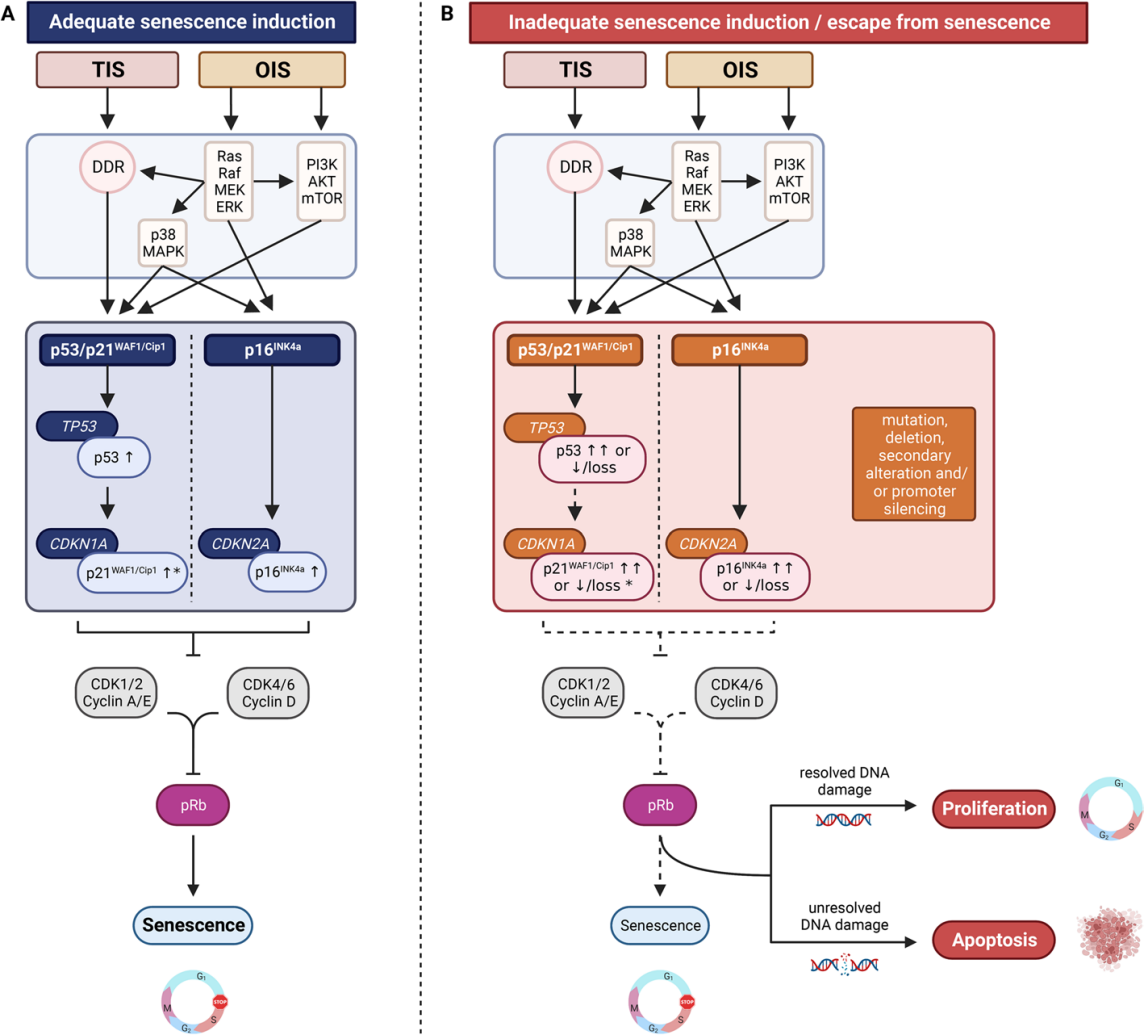
Molecular pathways of OIS and TIS. **A** Adequate senescence induction via participation of the DDR and the Ras-Raf-MEK-ERK, PI3K/AKT/mTOR and p38/MAPK signaling pathways resulting in functionally activated cell cycle inhibitor pathways (solid arrows) and upregulation of tumor suppressor proteins p53, p21^WAF1/Cip1^ and p16^INK4a^. Functional p21^WAF1/Cip1^ and/or p16^INK4a^ induce a stable cell cycle arrest by inhibition of CDK (i.e., CDK1, CDK2, CDK4 and CDK6)—cyclin (i.e., cyclin A, E and D) complexes, thereby preventing phosphorylation of the retinoblastoma protein (solid inhibitor lines), which blocks S-phase entry and induces senescence (solid arrow). **B** Inadequate senescence induction or escape from senescence due to (acquired) mutations, deletions, secondary alterations and/or promoter silencing affecting cellular control genes *TP53* (encoding p53), *CDKN1A* (encoding p21^WAF1/Cip1^) *CDKN2A* (encoding p16^INK4a^), resulting in absent or dysfunctional cell cycle inhibitor pathway activation (dotted arrow) and absent or dysfunctional tumor suppressor proteins to induce or maintain senescence (dotted inhibitor lines and arrow). Depending on whether the DNA damage is repaired, the cell may resume proliferation or go into apoptosis. * p21^WAF1/Cip1^ can also be activated by pathways that are independent of p53 [125]. OIS, oncogene-induced senescence; TIS, therapy-induced senescence; DDR, DNA damage response; CDK, cyclin-dependent kinase; pRb, retinoblastoma protein

The senescence-associated cell cycle arrest is considered fundamentally tumor-suppressive and the induction occurs through the involvement of different signaling and downstream cell cycle inhibitor pathways. Genotoxic stress induced by anticancer therapies results in a DNA-damage response (DDR) which leads to p53 and p21^WAF1/Cip1^ activation whereas oncogenic signaling and tumor suppressor inactivation results in downstream activation of both p53/p21^WAF1/Cip1^ and p16^INK4a^ via participation of the DDR and the Ras-Raf-MEK-ERK, PI3K/AKT/mTOR and p38/MAPK signaling pathways [6, 7, 62]. As such, TIS is primarily induced through p53/p21^WAF1/Cip1^ pathway activation whereas OIS is induced through either p53/p21^WAF1/Cip1^ and/or p16^INK4a^ pathway activation (Fig. 2A). Upregulation of functional tumor suppressor proteins p53, p21^WAF1/Cip1^ and p16^INK4a^ inhibit downstream cyclin-dependent kinase (CDK)—cyclin complexes, such as CDK2—cyclin E and CDK CDK4/6—cyclin D, preventing phosphorylation of the retinoblastoma protein [7]. Hyperphosphorylation of this tumor suppressor protein blocks S-phase entry [120] and is responsible for the induction of senescence [6] (Fig. 2A). Of note, despite tumor suppressor proteins p53, p21^WAF1/Cip1^ and/or p16^INK4a^ are primarily involved in TIS and OIS (Table 1), senescence can be induced [121] as well as bypassed [7, 16, 122–124] independent of p53/p21^WAF/Cip1^ and/or p16^INK4a^ pathway activation and inactivation/abrogation, respectively. Also, p21^WAF1/Cip1^ can be activated by pathways that are independent of p53 [125].

**Table 1:** Prognostic implications of OIS and TIS based on available *ex* and in vivo evidence of cancer patients with solid tumors according to cancer type and senescence trigger

Malignancy	Sample specification	Senescence marker^a^	Type of senescence^b^	Prognostic implication	Reference
**NSCLC**	Resected NSCLC	Lipofuscin	CS	High expression associated with worse OS	[126]
		p21^WAF1/Cip1^, cyclin E and Ki67			[127]
	Resected NSCLC SCC	p21^WAF1/Cip1^		Positive expression associated with improved survival	[128]
	Resected NSCLC AC / SCC			Reduced expression in stage III compared to stage I or II	
	NSCLC SCC	p16^INK4a^		High expression associated with improved OS	[129]
	Resected NSCLC AC / SCC			Loss of expression associated with worse OS	[130]
		macroH2A1.1		Low expression associated with worse DFS	[131]
	Resected NSCLC AC	Tumoral senescence signature (lipofuscin, p16^INK4a^, p21^WAF1/Cip1^ and Ki67^c^)		Tumoral senescence signature associated with worse DFS and OS	[132]
	Resected NSCLC AC / SCC / other histology		TIS: platinum-based CT with or without RT	Evidence of TIS as treatment outcome	
	NSCLC AC	Senescence-related gene signature: *FOXM1*, *HJURP*, *PKM*, *PTTG1* and *TACC3*	CS	High expression associated with: - disease progression and worse OS - immune-suppressive and protumorigenic TME - high expression of immune checkpoint genes and TMB levels	[133]
		Senescence marker genes: *VPS26A* and *LANCL1*		High expression associated with decreased survival	[134]
	NSCLC AC	Senescence marker genes: *VAMP3, STX4* and *ARMX3*		High expression associated with increased survival	
	NSCLC AC / SCC	Senescence marker genes: *NTAL, DEP1* and *B2MG*		High expression associated with mixed survival outcomes depending on histology	
	Resected NSCLC	SA-β-Gal and CDK1	TIS: CB/PTX	Expression demonstrates TIS as treatment outcome	[37]
		SA-β-Gal	TIS: CB/PTX or CRT^d^	Expression associated with worse OS	[135]
	NSCLC AC	CS gene score	CS	Lower CS gene score in primary tumors compared to adjacent normal solid tissue	[136]
	NSCLC SCC				
**Malignant pleural mesothelioma**	Resected MPM	SA-β-Gal, p21^WAF1/Cip1^ and PAI1 and mRNA p21^WAF1/Cip1^ and PAI1	TIS: platinum-based CT	- Increased expression after neoadjuvant CT - Stable disease associated with PAI1 expression, time-to-progression and worse OS	[137]
**Breast cancer**	Breast cancer	Senescence marker gene panel: *DEP1, NTAL, EBP50, STX4, VAMP3, ARMX3, B2MG, LANCL1, VPS26A* and *PLD3*	CS	High expression associated with increased OS	[134]
		CS gene score		Lower score in primary tumors compared to adjacent normal solid tissue	[136]
	Resected primary invasive ductal carcinoma	p16^INK4a^ and p53		Expression associated with worse DFS and OS	[138]
	Resected breast cancer	p16^INK4a^		Overexpression associated with unfavorable prognostic indicators (high grading, negative estrogen receptor status, inverse progesterone receptor status and high Ki67 expression) and indicative of a more undifferentiated malignant phenotype	[139]
		p21^WAF1/Cip1^ and p53		Low p21^WAF1/Cip1^ expression along with p53 overexpression associated with short DFS and OS, suggesting p53 overexpression reflects complete abrogation of p53 function	[140]
		p21^WAF1/Cip1^		Expression associated with worse DFS and OS	[141–143]
	Early breast cancer	uPA-PAI1		- High levels associated with worse DFS - Patients with high levels benefit more strongly from adjuvant CT	[144, 145]
	Resected triple negative breast cancer	macroH2A1.1 mRNA ratio		High ratio associated with worse DFS	[146]
	Resected breast cancer	SA-β-Gal	TIS: CP/DOX/5-FU	Expression demonstrated TIS as treatment outcome	[147]
		p21^WAF1/Cip1^, p27^Kip1^, p53 and cyclin D3	TIS: CP/MTX/5-FU or ECX/DOC		[148]
		p21^WAF1/Cip1^, H3K9Me3 and lamin B1^c^	TIS: DOC/DOX/CP, PTX/DOX/CP, DOX/CP, DOC/CP, 5-FU/EPX/CP or 5-FU/EPX/CP followed by DOC		[149]
		mRNA of p16^INK4a^, p21^WAF1/Cip1^ and CCNA1	TIS: EPX/CP	- High expression demonstrated TIS as treatment outcome - Persistent senescent cells evaded immune clearance	[150]
		Lipofuscin	CS	Expression present in TME	[151]
			TIS: CT^d^		
**Cervical, uterine, UCEC and ovarian cancer**	Normal cervical epithelium	p16^INK4a^, p21^WAF1/Cip1^, p15^INK4a^ and p14^ARF^	OIS	Almost completely negative expression	[152, 153]
	Cervical dysplastic and SCC			Overexpression	
	Normal cervical epithelium, CIN and cervical carcinoma	p21^WAF1/Cip1^		Higher expression in cervical carcinoma compared to normal cervical epithelium and CIN	[154]
				Expression associated with advanced stage	[155]
	Cervical AC			Expression associated with favorable prognosis	[156]
	Cervical SCC	CS gene score	CS	Positively correlated with PD-L1 protein expression and T cell cytotoxicity	[136]
	UCEC			Lower score in primary tumors compared to adjacent normal solid tissue	
	Resected pEOC	SA-β-Gal		Expression demonstrated spontaneous CS	[157]
		p21^WAF1/Cip1^ and p53		Low p21^WAF1/Cip1^ expression along with p53 overexpression associated with higher recurrence rate, suggesting p53 overexpression reflects complete abrogation of p53 function	[158]
	Resected HGSOC	SA-β-Gal and γH2AX		Higher expression in older patients	[159]
	Resected OC	p53, p16^INK4a^ and pRb		Negative or high p53, high p16^INK4a^ and reduced pRb expression associated with worse OS	[160]
	Primary OC	SA-β-Gal, p16^INK4a^, H1-β and Ki67^c^		Identification of senescent CAFs adjacent to epithelial ovarian cancer cells suggested to promote ovarian cancer tumorigenesis	[161]
	Normal ovary, primary OC, metastasis of OC, recurrent OC	p21^WAF1/Cip1^		- Expression gradually increased from normal ovary through primary OC, metastasis of OC and recurrent OC - Expression associated with decreased time-to-progression	[162]
	Advanced-stage serous OC and suboptimally debulked OC	Senescence marker genes: *VAMP3, ARMCX3* and *B2MG*	CS/TIS^d^	High expression associated with decreased survival	[134]
		Senescence marker genes: *EBP50* and *NTAL*		High expression associated with increased survival	
	Resected HGSOC	p16^INK4a^ and lamin B1^c^	TIS: CB/PTX	High expression associated with improved 5-year OS	[163]
**Esophageal cancer**	Precursor and ESCC lesions	Dec1	OIS	Expression demonstrated OIS as tumor-suppressive mechanism	[164]
	Resected ESCC			Low expression: - correlated with poor clinicopathological parameters (i.e., T-stage, lymph node metastasis and pathological TNM-stage) - associated with worse DFS	
		p16^INK4a^, p21^WAF1/Cip1^, pRb, Bax protein and cyclin D1		Combined high expression and low cyclin D1 expression associated with improved OS	[165]
	Normal tissue, precursor lesions and ESCC	p16^INK4a^, p14^ARF^ and p15^INK4b^		Increased expression in ESCC and in poorly differentiated specimens with lymph node metastasis, suggesting involvement of CS in cancer progression	[166]
		p21^WAF1/Cip1^		- Expression frequently found in precursor lesions and invasive ESCC compared to normal tissue - High expression associated with worse OS in curatively treated ESCC	[167]
	EC	Senescence gene signature: *ADH1B*, *IL1A*, *SERPINE1*, *SPARC*, *EZH2* and *TNFAIP2*		Enriched senescence gene signature in noncancerous cells of TME associated with improved OS	[168]
	ESCC	CS gene score	CS	Lower score in primary tumors compared to adjacent normal solid tissue	[136]
**Gastric cancer**	Resected gastric cancer	Senescence gene signature: *ADH1B*, *IL1A*, *SERPINE1*, *SPARC*, *EZH2* and *TNFAIP2*	CS	Enriched senescence gene signature in noncancerous cells of TME associated with: - improved DFS and OS - related to MSI, higher TMB and improved benefit from immunotherapy	[168]
		Senescore based on proteins ADH1B, IL1A, SERPINE1, SPARC, EZH2 and TNFAIP2		High-senescore protein expression associated with improved OS	
	Gastric AC	CS gene score		Lower score in primary tumors compared to adjacent normal solid tissue	[136]
	Gastric cancer	p21^WAF1/Cip1^ and mRNA of p21^WAF1/Cip1^		Increased expression associated with improved OS	[169]
	Primary gastric adenocarcinoma	p21^WAF1/Cip1^ and p53		Low p21^WAF1/Cip1^ expression along with p53 overexpression associated with more aggressive tumoral characteristics, higher recurrence rate and poorer survival, suggesting p53 overexpression reflects complete abrogation of p53 function	[170]
**Colorectal cancer**	*KRAS* and *BRAF* mutated benign serrated polyps	SA-β-Gal, p16^INK4a^ and Ki67^c^	OIS	- Expression demonstrated OIS - OIS increased with degree of dysplasia	[171, 172]
	Colon adenoma	SA-β-Gal, p16^INK4a^, p53, HP1α, HP1γ, H3K9me3 and Ki67^c^		Expression demonstrated OIS as tumor-suppressive mechanism	[15–17]
	Dysplastic aberrant crypt foci and adenomas	p21^WAF1/Cip1^		Lower expression suggesting dysregulated expression of cell-cycle controlling genes in tumorigenesis of CRC	[173]
	Early invasive colorectal carcinoma	SA-β-Gal, p16^INK4a^, p53, HP1α, HP1γ, H3K9me3 and Ki67^c^		Reduced or lost expression demonstrated loss of OIS	[15, 17, 171, 172]
	Resected primary CRC	p21^WAF1/Cip1^, NTAL, ARMCX3, EBP50 and γH2AX	CS	- Absent and extensive expression associated with negative prognosis, moderate expression with best prognosis - Distance between senescent cells and CD8^+^ T cells and a higher % CD8^+^ T cells near senescent cells linked to increased DSS and PFS, suggesting tumor-suppressive potential of CS is determined by TME and immune cell-mediated elimination of senescent tumor cells	[174]
		p16^INK4a^ and Ki67^c^		- Intratumoral senescence (high p16^INK4a^ and low Ki67) associated with reduced T cell infiltrates and low-grade inflammatory cell infiltrate - Low p16^INK4a^ expression associated with decreased survival	[175]
	CRC	Senescence marker genes: *VPS26A, ARMCX3* and *B2MG*		High expression associated with decreased survival	[134]
		Senescence marker genes: *NTAL*		High expression associated with increased survival	
		Senescence gene signature: *ADH1B*, *IL1A*, *SERPINE1*, *SPARC*, *EZH2* and *TNFAIP2*		Enriched senescence gene signature in noncancerous cells of TME associated with improved OS	[168]
	Metastasized CRC	p-ERK, HP1γ and PAI1	TIS: 5-FU/leucovorin	- Expression demonstrated TIS - TIS associated with longer PFS	[176]
	Resected CRC	SA-β-Gal and mRNA of p21^WAF1/Cip1^, p16^INK4a^ and IL-8	TIS: 5-FU and concomitant RT	- Increased expression demonstrated TIS - TIS increased rectal cancer invasiveness by upregulation of EMT related genes	[177]
	CRC	p21^WAF1/Cip1^	TIS: bevacizumab-based CT	- Increased expression demonstrated TIS - TIS associated with longer PFS	[178]
	CRC AC		CS	Decreased expression associated with higher Dukes stage, metastasis and worse survival	[179]
	CRC			Downregulation and negative expression associated with MSI	[180, 181]
				Decreased expression associated with lymph node and/or liver metastasis and worse survival	[182, 183]
		p53		Absent expression associated with MSI	[181]
	CRC AC	CS gene score		Lower score in primary tumors compared to adjacent normal solid tissue	[136]
	Rectal AC				
	Rectal cancer	p21^WAF1/Cip1^	TIS: concurrent CRT (5-FU alone or with OXP)	Increased expression demonstrated TIS of inflammatory CAFs with a pronounced stromal response	[184]
		Senescence gene signature			
	PC caused by cancer of the appendix, colon, rectosigmoid or rectum	SA-β-Gal, p16^INK4a^, p21^WAF1/Cip1^, H3K9me3 and Ki67^c^	OIS	- Increased expression demonstrated that PC is characterized by senescent tumor cells, and showed features of stemness - Low or absent SA-β-Gal expression in primary tumor and liver metastasis samples compared to high SA-β-Gal expression in PC samples, suggesting that the peritoneal cavity is a metastatic niche that induces senescence, whereas no signs of senescence induction within the metastatic environment of the liver - Absent SA-β-Gal expression in TILs of primary tumor and liver metastasis samples compared to elevated SA-β-Gal expression in TILs of PC samples, suggesting that senescent PC cells induce senescence in TILs	[185]
		Senescence-associated and SASP genes		Upregulated in PC compared to primary tumor samples, and have a distinct SASP, demonstrating that senescent PC with stem cell-like features express a unique SASP	
**Pancreatic cancer**	Resected PanIN and PDAC	p16^INK4a^ and Ki67^c^	OIS	High expression in low-grade PanINs, no expression in PDAC	[186]
		p21^WAF1/Cip1^		Expression increased with increasing grade of malignancy, demonstrating that aberrant cell cycle regulatory genes may be important in early development and progression of PanIN	[187]
	Precursor lesions (acinar to ductal metaplasia), PanINs and PDAC	SA-β-Gal		Expression only in precursor lesions (acinar to ductal metaplasia) and PanINs, no expression in PDAC	[188]
	Normal pancreas, pancreatitis and PDAC	SIN3B		- Absent or low expression in control pancreas and PDAC - Strong expression in pancreatitis and PanINs and correlated with IL-1α	[189]
**Hepatocellular carcinoma**	Cirrhosis, dysplasia and HCC	Senescence-related genes: *FAM38D*, *ATAD2*, *TOP2A*, *CCNE2*, *CRNDE*, *EPCAM*, *TMEM27*, *TFPI2*, *FOS*, *NAT2*, *GPR128*, *CYP39A1*, *FAM134B*, *SDCBP2* and *MUM1L1*	CS	Increased expression in liver cirrhosis, dysplasia being a transitional state to HCC and HCC that displayed immortal gene expression phenotypes	[190]
	Chronic hepatitis C and cirrhosis	SA-β-Gal		Expression correlated with fibrosis progression in cirrhosis and chronic hepatitis C, suggesting CS predispose to HCC development	[191, 192]
		p21^WAF1/Cip1^		Expression higher in cirrhosis compared to chronic hepatitis and associated with HCC development, suggesting p21^WAF1/Cip1^-related tumorigenesis in HCC	[193]
	Biliary cirrhosis	p21^WAF1/Cip1^/Ki67^c^ ratio		Increased expression ratios	[194]
	Cirrhosis and HCC	p21^WAF1/Cip1^ and p16^INK4a^		Increased expression in cirrhosis, strongly reduced in HCC	[195]
	Normal, chronic hepatitis C and HCC	SA-β-Gal		- Expression gradually increased from normal through chronic hepatitis C samples and HCC - Expression in non-tumoral liver tissue correlated with HCC in surrounding liver	[192]
	HCC	CS gene score		Lower score in primary tumors compared to adjacent normal solid tissue	[136]
	Peritumoral HCC tissue	Senescence-related gene signature: *FAM38D*, *ATAD2*, *TOP2A*, *CCNE2*, *CRNDE*, *EPCAM*, *TMEM27*, *TFPI2*, *FOS*, *NAT2*, *GPR128*, *CYP39A1*, *FAM134B*, *SDCBP2* and *MUM1L1*	OIS	Presence associated with: - early recurrence and poor survival - associated with chemokine (CCL2, CCL5 and CXCL11) and myeloid-specific gene expression and depletion in NK cell-specific gene activity	[196]
		p16^INK4a^ and p21^WAF1/Cip1^		Expression correlated with increased presence of CCR2 + myeloid cells	
	HCC	Senescence-related genes: *LRP4, OPRK1, PRAC2, N4BP3, GAL, CORO2B, FZD7, SEPTIN3, SMOX, EPO, MSC, GLP1R, HOXC6, PAPPA2, STK39, DLGAP5, THEM64, UNC5B, SLC16A11, CDH1, PRR15L, CCDC146, FAM117A, SLC2A4, CD2* and *STAT4*		High senescence score: - negatively correlated with the infiltration level of immunostimulating cells (plasma cells, CD8 T cells, activated CD4 memory T cells, gamma delta T cells and M1 macrophages) and positively correlated with the infiltration of immune-suppressive cells (memory B cells, naive CD4 T cells, M0 macrophages, M2 macrophages and eosinophils) - negatively correlated with the expression levels of immune checkpoint related genes (i.e., *CD274, LAG3, PDCD1L,* *SIGLE* and *TIFIT*) and lower response rate to immunotherapy - related to immune dessert subtype of HCC - associated with worse survival	[197]
		Senescence marker protein 30		- Decreased expression in adjacent non-tumor tissue, larger tumor size and enhanced TMN-stage - Decreased expression associated with worse OS	[198]
**Cholangiocarcinoma**	Premalignant bile duct adenomas, ductular reactions and CCA	p16^INK4a^	OIS	Expression in most premalignant bile duct adenomas and ductular reactions whereas barely expression in CCA, demonstrating OIS as tumor-suppressive mechanism	[199–201]
	CCA	CS gene score	CS	Lower score in primary tumors compared to adjacent normal solid tissue	[136]
**Prostate cancer**	Prostate IN	SA-β-Gal and CXCR2	CS	Expression demonstrates CS as tumor-suppressive mechanism	[122]
	BPH and prostate IN	SA-β-Gal, HP1α and HP1γ			[14, 18]
	Primary prostate cancer	GLB1		Expression associated with: - favorable clinicopathologic features (T stage and non-metastatic samples) - improved prostate specific antigen-free survival	[202]
	Resected prostate cancer		TIS: ADT^d^	Increased expression in tissues undergoing ADT longer than 5 months and in clinically more favorable intermediate grade cancers, demonstrating TIS as treatment outcome	[203]
		GLB1, HP1γ and Ki67^c^		Increased expression suggests TIS might be responsible for incomplete tumor regression	[204]
		mRNA of p16^INK4a^ and p21^WAF1/Cip1^	TIS: MIT	Increased expression and expression of a SASP (increased mRNA levels encoding IL-6, IL-8, GM-CSF, GRO-α, IGFBP-2, and IL-1β), demonstrating TIS with SASP expression as treatment outcome	[73]
		mRNA of p16^INK4a^, p21^WAF1/Cip1^ and CCNA1	TIS: MIT and MIT/DOC	- High expression demonstrated TIS as treatment outcome - Persistent senescent cells evaded immune clearance	[150]
		p21^WAF1/Cip1^	CS	Increased expression associated with high Gleason score and worse survival	[205, 206]
			TIS: ADT^d^	- Increased expression associated with p53 accumulation after ADT, suggesting TIS as treatment outcome - Increased expression associated with worse survival	[206]
	Prostate AC	CS gene score	CS	- Lower score in primary tumors compared to adjacent normal solid tissue - Positively correlated with PD-L1 protein expression and T cell cytotoxicity - High score associated with improved PFS and OS - Lower score associated with higher Gleason score, T and N stages - Predicted active immune response and better prognosis	[136]
**Bladder cancer**	Precancerous and cancerous urinary bladder	p16^INK4a^, HP1α, HP1γ and H3K9me3	OIS	Expression demonstrated OIS as tumor-suppressive mechanism in precancerous and cancerous lesions	[17]
	Radical cystectomy or transurethral resection	p16^INK4a^, p21^WAF1/Cip1^, p53 and pRb		Aberrant individual and/or combined expression associated with recurrence and worse OS	[207]
	Transitional cell carcinoma	p21^WAF1/Cip1^		- Expression associated with worse DFS and OS in superficial lesions - Loss of expression associated with worse DFS and OS in invasive lesions when accompanied by p53 accumulation	[208]
	Bladder urothelial carcinoma	CS gene score	CS	Lower score in primary tumors compared to adjacent normal solid tissue	[136]
**Skin cancer**	Human benign naevi	SA-β-Gal and p16^INK4a^	OIS	Increased expression demonstrated p16^INK4a^-dependent OIS as tumor-suppressive mechanism	[13, 209]
	Dermal neurofibroma				[210]
	Dysplastic naevi and radial early melanoma	p16^INK4a^, p53 and p21^WAF1/Cip1^		Less p16^INK4a^ expression and some p53 and p21^WAF1/Cip1^ expression demonstrated p53-dependent OIS as tumor-suppressive mechanism	[209]
	Advanced melanoma			No p16^INK4a^ and p21^WAF1/Cip1^ expression in advanced melanomas demonstrated escape from p16^INK4a^-dependent and/or p53-dependent OIS	
	Benign melanocytic and dysplastic naevi, in situ, invasive and metastatic melanoma	p16^INK4a^		Expression gradually decreased with increasing grade of malignancy	[211, 212]
	Primary melanoma			No association with DFS or OS	[212]
	Cutaneous malignant melanoma			Loss of expression correlated with tumor cell proliferation, thicker lesions and invasive stage	[213, 214]
	Vertical growth phase melanoma			Loss of expression associated increased tumor cell proliferation and poor prognosis	[215]
	Aggressive nodular malignant melanoma			Loss of expression associated with recurrent disease	[216]
	Melanoma	CS gene score	CS	- Positively correlated with PD-L1 protein expression and T cell cytotoxicity - Exhibited higher AUCs than the TIDE score for predicting immunotherapy response	[136]
	Basal cell carcinoma			CS gene scores of malignant cells from non-responders significantly decreased after treatment whereas posttreatment CS scores significantly increased in ICB responders	
	Merkel cell carcinoma				
**Thyroid cancer**	Early-stage papillary thyroid microcarcinoma, PTC and anaplastic thyroid carcinoma	p16^INK4a^, p21^WAF1/Cip1^ and IGFBP7	OIS	Expression gradually decreased with increasing grade of malignancy indicating involvement of OIS in thyroid carcinogenesis	[217]
	^*V600E*^*BRAF* PTC	SA-β-Gal and p16^INK4a^		Expression next to proliferating cancer cells demonstrating OIS and cells escaping from OIS co-exist in ^*V600E*^*BRAF* PTC	[218]
	(^*V600E*^*BRAF*) PTC	SA-β-Gal, p16^INK4a^, and Ki67^c^ and mRNA of p16^INK4a^		- Senescent tumor cells frequently present at invasive borders with features of collective invasion and high invasive ability with expression of a SASP - Senescent tumor cells existed during lymphovascular invasion and metastasis - Increased expression of CXCL12 in presence of senescent tumor cells in collective invasion area and diffuse CXCR4 expression in all PTC, demonstrating senescent tumor cell involvement in collective invasion and metastasis of PTC	[219]
**Bone and soft tissue tumors**	High grade sarcoma	p16^INK4a^	OIS	Decreased expression	[220]
	Dedifferentiated liposarcoma, synovial sarcoma and leiomyosarcoma			Decreased expression associated with reduced survival indicating p16^INK4a^-dependent OIS as tumor-suppressive mechanism	
	Liposarcoma	Senescence marker genes: *VPS26A* and *VAMP3*		High expression associated with increased survival	[134]
		Senescence marker genes: *STX4*		High expression associated with decreased survival	
	Osteosarcoma	Aging-/senescence-induced gene risk score		High score associated with: - worse OS - higher copy number variation score, implying a higher degree of tumor cell malignancy - immune cold tumors: lack of innate immune activation of infiltrating immune cells, less infiltration of antigen presenting cells, high TIDE score and T cell rejection - higher exhaustion and T cell proliferation scores - enriched MIF, CLEC and VEGF signaling pathway which are involved in osteosarcoma growth and metastasis and blood vessel growth	[221]
**Renal cell carcinoma**	Primary RCC	p400	OIS	Decreased expression associated with advanced tumor stage, higher grade of malignancy, regional lymph node metastasis and poor prognosis	[222]
	RCC	SA-β-Gal, p53, Dec1, Ki67^c^ and Raf-1^c^	TIS: sunitinib	Increased expression demonstrates TIS as treatment outcome	[223]
		CS gene score	CS	- Lower score in primary tumors compared to adjacent normal solid tissue - Exhibited higher AUCs than the TIDE score for predicting immunotherapy response	[136]
	KIRP				
**Brain malignancies**	PA	SA-β-Gal, p16^INK4a^, p53 and Ki67^c^	OIS	Identification of OIS responsible for slow growth pattern, lack of progression to higher-grade tumors and high OS	[224]
		Senescence-associated genes: *CDKN2A, CDKN1A, CEBPB, GADD45A, and IGFBP7*			
		SASP factors: FGF2, IL-15, CSF3, VEGFA, IL-17A, CCL2, CXCL8, CSF2, CCL3, IFNγ, IL-6, IL-13, CCL11 and IL-1β		High expression and upregulation of SASP associated with favorable PFS, demonstrating OIS is regulated by SASP	[225]
	High-grade tumors and adult LGG	*CDKN2A* and *TP53*		Homogeneous deletion of *CDKN2A* and secondary alterations of *CDKN2A* and *TP53* more common	[226–228]
	IDH-mutant and lower grade (WHO grade II-III) astrocytoma			Absence of mutations associated with increased survival	[229, 230]
	ACP	p21^WAF1/Cip1^, p53, GLB1, γH2AX, phosphor-DNA-PKc and Ki67^c^		Identification of senescent cells harboring molecular signature of OIS and SASP, demonstrating OIS and SASP drive cell transformation and tumor initiation	[231]
		SASP factors: IL-1β, IL-6, IL-8, IL-10, IL-18, TNFα and IFNγ		Identification of SASP, demonstrating OIS and SASP drive cell transformation and tumor initiation	[232]
	Medulloblastoma	*CDKN2A* and p53 pathway		Frequent *CDKN2A* promoter methylation and p53 pathway mutations, demonstrating OIS escape underlies tumor progression	[233–235]
	Low-grade diffuse astrocytoma	p16^INK4a^ and pRb		Loss of expression associated with shorter survival	[236]
	Glioma	Senescence score based on senescence-associated genes: *CCL2, CCL7, CDKN1A, COPG, CSF2RB, CXCL1, ICAM-1, IGFBP-3, IL-6, IL-8, SAA4, TNFRSF-11B, TNFSF-11 and TP53*	CS	Senescence score: - associated with poor prognosis - correlated with older age - increases with WHO histological grade (lowest values for low-grade astrocytomas (WHO II), higher values for anaplastic astrocytomas (WHO III) and highest values for glioblastomas (WHO IV) and gliosarcomas), linking senescence-associated gene signature to disease progression	[237]
		SA-β-Gal and Ki67^c^		Identification of senescent cells	[238]
		Senescence gene signature: *ANXA5*, *APOE*, *CD151*, *CDKN1A*, *CDKN2A*, *CDKN2B*, *CTSB*, *CTSD*, *CTSL*, *CTSZ*, *EMP3*, *FTH1*, *LFITM3*, *LGFBP2*, *LGFBP3*, *LAMP1*, *LAMP2*, *LGALS1*, *MT1*, *OCIAD2*, *PDLIM4*, *RBP1*, *S100A11*, *SEP11*, *SDC4*, *SPARC*, *TIMP1*, *TM4SF1*, *TMSB4X*, *TNC* and *TNFRSF12A*		Senescence gene signature associated with shorter survival	
	Normal and reactive brain tissue and glioma	p21^WAF1/Cip1^		Increased expression in glioma compared to normal and reactive brain tissue, suggesting p21^WAF1/Cip1^-related tumorigenesis in glioma	[239]
	Astrocytic glioma			Expression associated with worse DFS	[240]
	Astrocytic and high-grade glioma	Senescence marker genes: *NTAL* and *STX*		High expression associated with decreased survival	[134]
	Astrocytic and high-grade glioma and glioblastoma	Senescence marker genes: *VPS26A, ARMCX3* and *B2MG*		High expression associated with increased survival	
	Glioblastoma multiforme	CS gene score	CS	Positively correlated with PD-L1 protein expression and T cell cytotoxicity	[136]
**Head and neck cancer**	Oropharyngeal SCC	p16^INK4a^	OIS	Expression associated with favorable prognosis regardless of HPV status	[241]
	Laryngeal, hypopharyngeal or oral SCC			No prognostic value	[242]
	Normal, benign hyperplastic skin and oral lesions			Negative expression	[243, 244]
	dysplastic and carcinoma in situ skin and oral SCC			Heterogeneous expression	
	Advanced skin and oral SCC			Consistent expression at areas of microinvasion and at superficial margins	
	deeply invasive skin and oral SCC			Near to complete absent expression, demonstrating p16^INK4a^-dependent OIS as tumor-suppressive mechanism	
	Oral SCC	p16^INK4a^, p53, pRb and cyclin D1		- Loss of p16^INK4a^ expression earliest event in tumorigenesis - Deregulation of pRb and p53 associated with malignant transformation and adverse prognosis	[245]
	Premalignant dysplastic and SCC of skin and oral epithelium and HNSCC	*CDKN2A*		High frequency of gene mutation, deletion and promoter silencing	[243, 246]
	HNSCC	H3K9Me		- Identification of senescent cells in 67.1% of biopsies - More senescent cells in tumor center compared to invasive front - No prognostic impact	[247]
		Senescence score based on senescence-associated genes: *DUSP16*, *EHF*, *ITSN2*, *DUSP3*, *HDAC4*, *TXNIP*, *KL*, *MAP4KI*, *PIK3R5*, *YPEL3*, *CDKN2A*, *MAP2K7*, *PIAS4*, *POU5F1*, *EZH2*, *DGCR8*, *TYK2*, *BTG3*, *SOCS1*, *G6PD*, *TXN*, *DPY30*, *AURKA*, PDCD10, *PSMD14*, *FXR1*, *PCGF2*, *GAPDH*, *PSMB5*, *RSL1D1*, *IL1A*, *CDK6*, *LIMA1*, *CAV1*, *SERPINE1*, *HSPA5*, *NEK6*, *ASPH*, *MAP2K1*, *ACLY*, *TOP1*		High-senescence score: - associated with worse OS - correlated to poor clinicopathological parameters (histologic grade, TNM-stage, T-stage and lymph node metastasis)	[248]
	Pharyngeal and laryngeal HNSCC	p21^WAF1/Cip1^		High expression associated with poor OS	[249]
	Oral SCC				[250]
	Tonsillar SCC			Expression associated with favorable DSS	[251]
	Laryngeal and oral HNSCC			Higher expression associated with improved OS in stage III patients	[252]
	Minor salivary gland adenoid cystic carcinoma	p16^INK4a^ and p21^WAF1/Cip1^	CS	Strong and intense p21^WAF1/Cip1^ expression and complete negative p16^INK4a^ expression, suggesting transient senescence insufficient to maintain the senescence-associated cell cycle arrest, avoiding cell death by senescence and favoring tumor growth	[253]
	HNSCC	H3K9Me	TIS: RT with or without 5-FU/CP or 5-FU/CB	- Less senescent cells in post-RCT samples - No prognostic impact	[247]
		mRNA of CXCR2 receptor and/or its ligands (CXCL1–3, CXCL5, CXCL7, and CXCL8)	TIS: CRT^d^	Increased expression associated with impaired DSS, demonstrating TIS and SASP production determines radioresistance	[254]
		CS gene score	CS	Lower score in primary tumors compared to adjacent normal solid tissue	[136]
**Thymic cancer**	Thymic cancer	CS gene score	CS	Positively correlated with PD-L1 protein expression and T cell cytotoxicity	[136]

^a^detected by immunohistochemistry unless otherwise specified

^b^defined as CS unless otherwise specified

^c^negative marker of senescence

^d^not further specified

*5-FU* 5-fluorouracil, *AC* Adenocarcinoma, *ACP* Adamantinomatous craniopharyngioma, *ADT* Androgen deprivation therapy, *AUCs* Areas under the curve, *BPH* Benign prostate hypertrophy, *CAFs* Cancer-associated fibroblasts, *CB* Carboplatin, *CCA* Cholangiocarcinoma, *CIN* Cervical intraepithelial neoplasia, *CLEC* C-type lectin-like, *CP* Cisplatin, *CP* Cyclophosphamide, *CRC* Colorectal cancer, *CRT* chemoradiotherapy, *CS* Cellular senescence, *CT* chemotherapy, *DFS* Disease-free survival, *DOC* Docetaxel, *DOX* Doxorubicin, *DSS* Disease-specific survival, *EC* Esophageal cancer, *ECM* Extracellular matrix, *ECX* Epirubicin, *EMT* Epithelial-mesenchymal transition, *ESCC* Esophageal squamous cell cancer, *HCC* Hepatocellular carcinoma, *HGSOC* High-grade serous ovarian cancer, *HNSCC* Head and neck squamous cell carcinoma, *ICB* Immune-checkpoint blockade, *IN* intraepithelial neoplasia, *KIRP* Kidney renal papillary carcinoma, *LLG* Low-grade glioma, *MIF* Macrophage migration inhibitory factor, *MIT* Mitoxantrone, *MPM* Malignant pleural mesothelioma, *MSI* Microsatellite instability, *MTX* Methotrexate, *NSCLC* Non-small cell lung cancer, *OC* Ovarian cancer, *OIS* Oncogene-induced senescence, *OS* Overall survival, *OXP* Oxaliplatin, *PA* Pilocytic astrocytoma, *PanIN* Pancreatic intraepithelial neoplasia, *PC* Peritoneal cancer, *PD-L1* Programmed death-ligand 1, *PDAC* Pancreatic ductal adenocarcinoma, *pEOC* Primary epithelial ovarian cancer, *PFS* Progression-free survival, *pRb* Retinoblastoma protein, *PTC* Papillary thyroid cancer, *PTX* pPaclitaxel, *RCC* Renal cell carcinoma, *RCT* Radiochemotherapy, *RT* Radiotherapy, *SASP* Senescence-associated secretory phenotype, *SCC* Squamous cell carcinoma, *SS* Senescence signature, *TIDE* Tumor immune dysfunction and exclusion, *TILs* Tumor-infiltrating lymphocytes, *TIS* Therapy-induced senescence, *TMB* Tumor mutational burden, *TME* Tumor microenvironment, *UCEC* Uterine corpus endometrial carcinoma, *VEGF* Vascular endothelial growth factor, *WHO* World Health Organization

There is abundant *ex* and in vivo evidence in several tumor types that OIS acts as a tumor-suppressive mechanism preventing the expansion of pre- or fully malignant cells (Table 1). OIS is found in precursor lesions and in low TNM stage tumors with more favorable clinicopathologic features [13, 202, 209], whereas in full-blown malignant lesions OIS-related markers are often dysregulated or completely lost [173] and correlate with higher TNM stage tumors and poor clinicopathological parameters [136, 164, 211, 212] (Table 1). Tumor suppressor protein p16^INK4a^ often comes forward as the main regulator for maintaining the OIS-associated cell cycle arrest which is considered to be more crucial for maintaining the senescence-associated cell cycle arrest whereas p53/p21^WAF1/Cip1^ pathway activation is more involved in the initiation of senescence [59]. Mutations, deletions, secondary alterations and/or promoter silencing of cellular control genes (i.e., *TP53*, *CDKN1A* and *CDKN2A*) encoding for tumor suppressor proteins p53, p21^WAF1/Cip1^ and p16^INK4a^ may result in inadequate senescence induction or escape from senescence due to absent or dysfunctional cell cycle inhibitor pathway activation and absent or dysfunctional tumor suppressor proteins to induce or maintain OIS. Hence, dysregulated (i.e., decreased or overexpressed) expression or complete loss of tumor suppressor proteins p16^INK4a^, p21^WAF1/Cip1^ and p53 are often correlated with increasing grade of malignancy and tumor progression, and associated with a negative prognostic outcome [139, 140, 158, 170, 187, 205–207, 213, 214, 236] (Table 1) (Fig. 2B).

However, in certain tumor types, the (abundant) presence of OIS or expression of senescence-associated markers is also linked to worse prognosis [132, 197, 221, 237, 238, 248]. Perhaps even more surprisingly, both absence and extensive presence of senescence in CRC was associated with negative prognosis whereas moderate presence was associated with the best prognosis [174], demonstrating that an extensive senescence burden can paradoxically impair clinical outcome in contrast to a moderate senescence burden.

Concerning TIS, evidence demonstrates that TIS is an in vivo relevant outcome of various anticancer therapies in several tumor types (Table 1). For example, in breast, colorectal and prostate cancer TIS was observed after neoadjuvant genotoxic chemotherapy [150, 177, 178] and antihormone therapy [204]. Therapy-induced senescent cells were identified in residual drug-resistant tumors [150] and in samples with partial or incomplete pathological response to neoadjuvant therapy [149], suggesting TIS might persist after neoadjuvant therapy [203] and is responsible for incomplete tumor regression [204]. The presence of TIS is however linked to contradictory clinical outcomes and is associated with worse [135, 137] as well as improved [163, 176, 178] prognosis depending on tumor type. For example, while in non-small cell lung cancer (NSCLC) TIS is associated with worse OS [135], in CRC a higher proportion of therapy-induced senescent cells after chemotherapy treatment was associated with a longer progression-free survival (PFS) compared to when the proportion of senescent tumor cells did not change before and after chemotherapy [178]. Thus, regardless of the type of cancer, the senescence burden of OIS and TIS seems to be an important determinant affecting the outcome in cancer patients.

#### Secretion, composition and time-dependent impact of SASP

Whereas the senescence-associated cell cycle arrest acts tumor-suppressive, SASP factors secreted by senescent cells can be both tumor-suppressive and tumor-promoting [255]. The main signaling pathways involved in SASP regulation include NF-κB, p38, mTOR, C/EBPβ and JAK2/STAT3 [16, 256–260]. Interleukin (IL)-1α is secreted by oncogene-induced and therapy-induced senescent cells and initiates the production of key SASP proteins such as IL-6 and IL-8 through activation of NF-κB and C/EBPβ [62, 261]. The senescent phenotype is subsequently enforced autocrinally by IL-6 [16] and IL-8 [122] and transmitted paracrinally to neighboring cells by IL-1α [262], further enhancing the production of these SASP factors. Abundant SASP factors IL-6 and IL-8 have both anti-tumorigenic and pro-tumorigenic effects [263]. For example, both interleukins mediate the recruitment of macrophages, T cells and natural killer (NK) cells supporting immune surveillance and elimination of senescent cancer cells [32] but also create a chronic inflammatory TME driving cancer development [73] and attract myeloid derived suppressor cells that suppress T [264] and NK cells [196] and blocks IL-1α signaling, preventing paracrine senescence in neighboring cancer cells [265]. Next to pro-inflammatory cytokines, the SASP may consist of a variety of chemokines (e.g., CCL2 and CXCL1), angiogenic factors (e.g., VEGF), growth factors (e.g., HGF, PDGF, EGF and TGFα), matrix-remodeling enzymes (e.g., MMP1 and MMP3) and bioactive lipids [62, 263]. However, its composition is highly dynamic [263], complex and variable and depend on the cell type, senescence-inducing trigger and type of senescence [62, 74, 75], resulting in cancer-specific and context-dependent effective SASP levels. Besides its variable composition, the SASP is suggested to have a time-dependent impact [31]. Whereas the short term presence of SASP is suggested to be primarily tumor-suppressive, the long term presence of pro-inflammatory SASP factors can drive cancer [31, 32]. Thus, depending on the secretion, composition and the duration of its presence, the net effect of the SASP may be tumor-suppressive or tumor-promoting, thereby either enhancing or opposing the tumor-suppressive property of the senescence-associated cell cycle arrest.

Evidence from patients with various tumor types show that oncogene-induced senescent cells are capable of secreting a tumor-promoting and immune-suppressive SASP that is linked to impaired clinical outcome. NSCLC patients with an elevated senescence-related gene signature score overexpressed an immune-suppressive SASP and demonstrated decreased infiltration levels of cytotoxic T cells and NK cells and increased levels of immune-suppressive cells (i.e., neutrophils, cancer-associated fibroblasts, regulatory T cells, and resting NK cells), disease progression and worse OS [133]. The importance of the interaction between the SASP and immune surveillance of senescent tumor cells is further emphasized by the finding that, in CRC, both a lower average distance between senescent cells and T cells as well as a higher percentage of T cells near senescent cells were linked to improved survival, suggesting that the tumor-suppressive potential of cellular senescence is determined by the TME and immune cell-mediated elimination of senescent tumor cells [174]. The SASP of senescent cells can also direct neighboring cells and drive cell transformation and tumor initiation [231, 232] and mediate collective invasion and metastasis [219], as evidenced in adamantinomatous craniopharyngioma and papillary thyroid cancer. Also in precursor lesions of pancreatic ductal adenocarcinoma (PDAC) (i.e., pancreatitis and pancreatic intraepithelial neoplasias), a senescence-associated inflammatory SASP was linked to PDAC progression [189].

Increasing evidence demonstrates that also therapy-induced senescent cells can produce a tumor-promoting and immune-suppressive SASP that might impair clinical outcome. For example, in response to genotoxic chemotherapy, TIS and a protumorigenic SASP were observed in prostate cancer resection samples [73], and overexpression of SASP factor were associated with impaired outcome in head and neck squamous cell carcinoma patients [254] as early TIS and SASP production upon radiotherapy was demonstrated in a preclinical model. In an elucidative study, therapy-induced senescent cells of breast and prostate cancer patients were found to evade immune clearance by shedding of natural killer group 2D (NKG2D) ligands and paracrine suppression of NKG2D-receptor-mediated immunosurveillance [150]. Of importance, since TIS depends on p53/p21^WAF1/Cip1^ pathway activation (Fig. 2), the tumoral p53 status indirectly determines SASP production and outcome after treatment with senescence-inducing anticancer therapies. This was illustrated in an in vivo p53 wild-type breast cancer model where TIS was induced instead of cell death after chemotherapy treatment and resulted in minimal regression of the tumor and early relapse through the secretion of protumorigenic SASP [266]. Accordingly, breast cancer patients harboring a *TP53* mutation showed an improved response to anthracycline-based chemotherapy [267, 268].

In contrast, abundant evidence links both OIS [165, 202] and TIS [163, 176, 178] to improved outcome (Table 1). In this scenario, it is conceivable that oncogene-induced and therapy-induced senescent cells secrete moderate to low effective SASP levels and/or secrete a SASP with a net tumor-suppressive and immune-promoting effect. For example, in pilocytic astrocytoma (PA), a low grade glioma and most common brain tumor in children, SASP factors were upregulated, and high levels of IL-1β and SASP expression were associated with favorable PFS [224]. The SASP was therefore suggested to regulate OIS in PA [225] and held responsible for the slow growth pattern, the lack of progression to higher-grade astrocytomas and the high OS of affected patients [224]. In addition, no oncogene-induced or therapy-induced senescent cells were identified in chemotherapy-naïve and neoadjuvant chemotherapy treated breast cancer samples, suggesting tumoral senescent cells either were already cleared by the immune system or bypassed senescence [151].

#### Senescence in the TME

There is mounting evidence that senescence also occurs in the TME and has prognostic implications. In gastric cancer, CRC and esophageal cancer patients, an enriched senescence gene signature in noncancerous cells, but not in cancerous cells, of the TME (e.g., endothelial cells, enteroendocrine cells, macrophages and fibroblasts) resulted in a longer disease-free survival and OS [168]. In contrast, identification of senescent cancer-associated fibroblasts (CAFs) adjacent to epithelial ovarian cancer cells in ovarian cancer specimens were suggested to promote ovarian cancer tumorigenesis [161]. The presence of a senescence-associated gene signature in peritumoral tissue of hepatocellular carcinoma (HCC) patients was also associated with early recurrence and poor survival as peritumoral OIS induced an accumulation of C–C chemokine receptor 2^+^ myeloid cells through secretion of C–C motif chemokine ligand 2, resulting in NK cell inhibition and enhanced HCC growth [196]. Interestingly, in a murine rectal cancer and patient-derived tumor organoids model, IL-1α was found to predispose inflammatory CAFs to p53-mediated TIS upon irradiation, which in turn resulted in chemoradiotherapy resistance and disease progression through the secretion of cytokines and extracellular matrix constituents supporting the invasion and metastasis of cancer cells and counteracting the irradiation-induced tumor cell death [184]. Consistently, the presence of inflammatory CAFs in pre-therapeutic patient biopsies resulted in poor chemoradiotherapy response and low IL-1α receptor antagonist serum levels, which enhances IL-1 signaling and predisposes inflammatory CAFs to TIS, correlated with poor prognosis in rectal cancer patients [184].

Hence, cellular senescence is not solely limited to cancerous cells but also occurs in cells of the TME as well as of the immune system [269].

#### Model for differential prognostic outcomes of OIS and TIS in cancer patients.

**Fig. 3:**
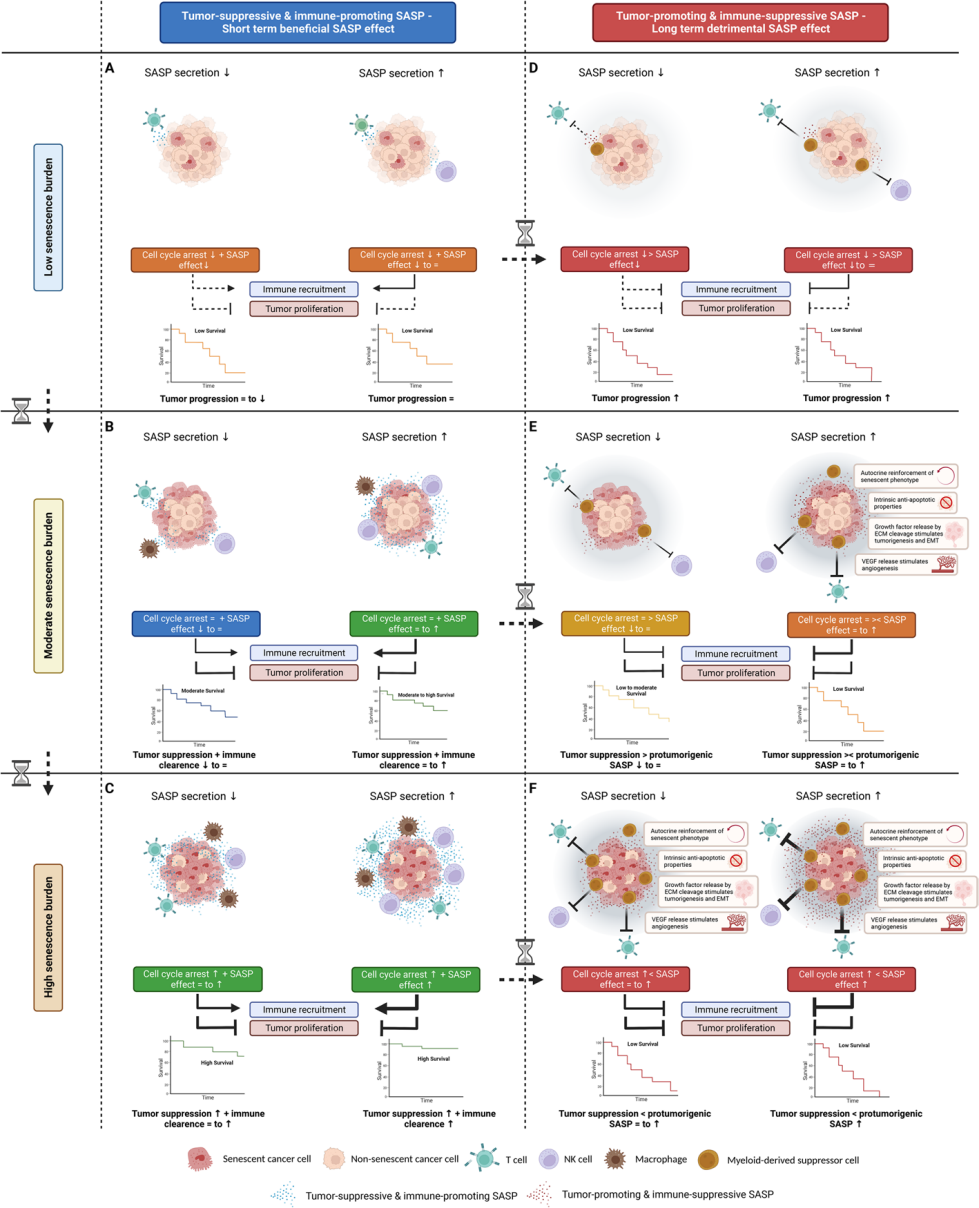
Model for differential prognostic outcomes of cellular senescence (OIS and TIS) in cancer patients. Tumor-suppressive and immune-promoting SASP—Short term beneficial SASP effect. In case of a net tumor-suppressive and immune-promoting SASP or short term presence of SASP, immune recruitment will result in immune clearance of senescent cancer cells as well as non-senescent cancer cells, thereby reinforcing cellular senescence to provide adequate tumor suppression. However, in case of a (**A**) low senescence burden, the effects of the SASP are expected to be less profound as the overall SASP levels secreted by the low number of tumoral senescent cells are lower compared to SASP levels in case of a moderate or high senescence burden. Therefore, the senescence-associated cell cycle arrest as well as the SASP levels are expected to be insufficient to provide an adequate tumor suppression. In case of a (**B**) moderate senescence burden, the senescence-associated cell cycle arrest can increasingly be reinforced in case of low and high SASP secretion, respectively, to provide adequate tumor suppression. In case of a (**C**) high senescence burden, the senescence-associated cell-cycle arrest can be reinforced by the tumor-suppressive and immune-promoting SASP in case of high as well as low SASP secretion due to the large number of tumoral senescent cells. As such, in case of a net tumor-suppressive and immune-promoting SASP, a high senescence burden result in improved outcome. Tumor-promoting and immune-suppressive SASP—Long term detrimental SASP effect. In case of a net tumor-promoting and immune-suppressive SASP or long term presence of SASP, the senescence-associated cell cycle arrest can be opposed by the SASP by molding an immune-suppressive and protumorigenic TME and stimulating immune evasion. However, in case of a **(D)** low senescence burden, the effects of the SASP are expected to be less profound as the overall SASP levels secreted by the low number of tumoral senescent cells are lower compared to SASP levels in case of a moderate or high senescence burden. Therefore, the senescence-associated cell cycle arrest in case of a low senescence burden is not opposed by the SASP, however, the senescence-associated cell cycle arrest is insufficient to prevent tumor proliferation. In case of a **(E)** moderate senescence burden, the senescence-associated cell cycle arrest can be opposed by the SASP in case of high SASP secretion, whereas in case of low SASP secretion the senescence-associated cell cycle arrest overrules the lower SASP levels, resulting in differential tumor-promoting and tumor-suppressive effects, respectively. In case of a **(F)** high senescence burden, the senescence-associated cell cycle arrest can be opposed and overruled by the protumorigenic effects of the SASP in case of high as well as low SASP secretion, as the overall SASP levels produced by the large number of tumoral senescent cells are elevated, even in case of low SASP secretion. As such, in case of a net tumor-promoting and immune-suppressive SASP, a high senescence burden can paradoxically result in worse outcome. ECM, extracellular matrix; EMT, epithelial-mesenchymal transition; VEGF, vascular endothelial growth factor; NK cell, natural killer cell; SASP, senescence-associated secretory phenotype; ↑, high; = , moderate; ↓, low; > , greater-than; < , less-than; ⌛, time

Evidence of several cancer types (presented in Table 1) suggests that the prognostic implications of OIS and TIS are highly context-dependent and primarily depend on the (i) the senescence burden; (ii) the secretion; and (iii) the composition of the SASP and/or duration of SASP presence. Therefore, in a simplified schematic model, we present different scenarios that could provide a rationale for the differential outcomes of cellular senescence observed in cancer patients, based on the interplay between these three factors, i.e., (i) the senescence burden (i.e., low, moderate or high); (ii) the secretion (i.e., low or high); and (iii) the composition of the SASP (i.e., net tumor-promoting and immune-suppressive or net tumor-suppressive and immune-promoting) and/or duration of SASP presence (i.e., short term or long term) (Fig. 3).

The senescence-associated cell cycle arrest is considered tumor-suppressive whereas the composition of the SASP and/or duration of SASP presence determines whether the senescence-associated cell cycle arrest is reinforced or opposed. The degree of SASP secretion and levels, which depend on the senescence burden, determines to which extent the senescence-associated cell cycle arrest is reinforced or opposed. As such, OIS and TIS can have tumor-suppressive and tumor-promoting properties (Fig. 3A-F).

The proposed model provides a rationale for the differential outcomes of OIS and TIS observed within the same cancer type, such as in [174], as well as between different types of cancer (Table 1). Accordingly, cancer cells originating from urinary systems, glands and soft tissues (e.g., prostate cancer, adenoid cystic carcinoma, RCC and melanoma) exhibited relatively higher cellular senescence gene scores than tumors originating from reproductive organs (e.g., breast cancer, cervical SCC and OC) [136], and were correlated with SASP factors and immune related genes, suggesting SASP-induced immune infiltration. The infiltration of immune cells varied however in a cancer-specific pattern [136], contributing to the context-dependency of the proposed model and the interplay between the senescence burden, the secretion and composition of the SASP.

Of note, as senescent cells can reinforce their senescent phenotype in an autocrine fashion [16, 122] and paracrinally transmit the senescent phenotype to adjacent malignant and non-malignant cells [262, 270], the tumoral senescence burden can increase over time resulting in altered tumoral repercussions (Fig. 3, vertical arrow hourglasses) in addition to the differential time-dependent impact of SASP (Fig. 3, horizontal arrow hourglasses). Thus, cellular senescence in cancer should be considered as a dynamic, rather than an irreversible, static condition [271], with antitumorigenic and protumorigenic features that can change over time.

## Conclusion

### Conclusions and future perspectives

Clinical evidence of cellular senescence in cancer patients has long been underestimated, in part due to the difficult detection, since currently no specific and universal markers for senescent cells exist. Historically, cellular senescence was primarily considered as an endogenous tumor suppressor mechanism halting the proliferation of damaged cells which are at risk of malignant transformation, thereby protecting against cancer. However, during the last two decades, a more nuanced view on the involvement of cellular senescence in tumorigenesis and response to therapy has emerged. Here, we provided a comprehensive overview on the prognostic implications of cellular senescence in cancer patients with solid tumors. Increasing clinical evidence add to the antagonistic pleiotropy of cellular senescence as differential prognostic outcomes, ranging from improved to impaired outcome, are demonstrated. In a simplified model we propose that the prognostic implications of OIS as well as TIS are highly context-dependent and primarily depend on the senescence burden, the secretion and the composition of the SASP and/or duration of SASP presence, thereby providing a rationale for the differential outcomes of OIS as well TIS observed within the same cancer type as well as between different types of cancer discussed in this review. However, (pre)clinical research is warranted to provide adequate evidence to further support this model, and to better comprehend when and how senescent cancer cells give rise to a beneficial or detrimental outcome.

The detection of cellular senescence in cancer patients can be achieved by various methods and using various markers. Despite clear algorithms to accurately assess and quantify senescent cells in vitro and in vivo [1, 44], a plethora of different senescence markers, single or combined with other markers, at different translational levels are currently used to demonstrate the presence of cellular senescence (Table 1). Hence, it is difficult to compare clinical data and to draw reliable conclusions regarding the prognostic implications of cellular senescence, as well as the implementation of emerging senolytics (i.e., targeted removal of senescent cells) [42, 78, 263, 271] and senomorphics that modify/suppress the SASP [32, 263, 272], underlining the need for a uniform and consistent application of recognized and validated markers of cellular senescence at different translational levels. Of note, as AI-based computational pathology is making its way into medicine and clinical practice [273], an AI-based detection of cellular senescence might potentially make multi-marker detection of senescent cells redundant in the near-future. Since the prognostic impact of senescence is mainly mediated by the SASP, extensive profiling of the SASP in specific disease contexts (i.e., organ- and trigger-specific (OIS versus TIS)), as well as the identification of biomarkers representing the senescence burden will be paramount [75]. Especially longitudinal monitoring of senescent cells and their SASP will be of particular interest, as preclinical models are not able to capture the beneficial or detrimental effects exhibited by senescent cells and the SASP over an extended period of time.

Since the TME, containing cancer-associated fibroblasts and infiltrating immune cells, is believed to be a major contributor to therapy resistance and disease progression [274], the interaction of TME with senescent cells as well as the SASP should be investigated more closely. This can be achieved using appropriate preclinical models that precisely recapitulate this complex heterogeneity, such as in vitro 3D culture technologies (e.g., organoids), thereby resembling a more physiological human cancer model [275]. Interestingly, by combining single-cell RNA-sequencing with spatial transcriptomics [276], it is feasible to map the location of distinct cell types and subpopulations in the TME and investigate the interaction of senescent cancer cells with the TME more in-depth.

As an emerging cancer hallmark, the involvement of cellular senescence in cancer is complex and highly context-dependent, exerting potential beneficial and/or detrimental effects. Therefore, senescence must be approached in a nuanced way regarding its repercussions in cancer. Only in this way it is possible to optimally exploit cellular senescence as an anticancer therapeutic strategy.

## Data Availability

Not applicable.

## Abbreviations

CDK: Cyclin-dependent kinase
CRC: Colorectal cancer
IL: Interleukin
NK: Natural killer
OIS: Oncogene-induced senescence
SA-β-Gal: Senescence-associated beta-galactosidase
SASP: Senescence-associated secretory phenotype
TIS: Therapy-induced senescence
TME: Tumor microenvironment

## References

[1] Gorgoulis V, Adams PD, Alimonti A, Bennett DC, Bischof O, Bishop C (2019). Cellular Senescence: Defining a Path Forward. Cell.

[2] Muñoz-Espín D, Cañamero M, Maraver A, Gómez-López G, Contreras J, Murillo-Cuesta S (2013). Programmed cell senescence during mammalian embryonic development. Cell.

[3] Storer M, Mas A, Robert-Moreno A, Pecoraro M, Ortells MC, Di Giacomo V (2013). Senescence is a developmental mechanism that contributes to embryonic growth and patterning. Cell.

[4] Demaria M, Ohtani N, Youssef SA, Rodier F, Toussaint W, Mitchell JR (2014). An essential role for senescent cells in optimal wound healing through secretion of PDGF-AA. Dev Cell.

[5] Jun JI, Lau LF (2010). The matricellular protein CCN1 induces fibroblast senescence and restricts fibrosis in cutaneous wound healing. Nat Cell Biol.

[6] Muñoz-Espín D, Serrano M (2014). Cellular senescence: from physiology to pathology. Nat Rev Mol Cell Biol.

[7] Paez-Ribes M, González-Gualda E, Doherty GJ, Muñoz-Espín D (2019). Targeting senescent cells in translational medicine. EMBO Mol Med.

[8] Hayflick L, Moorhead PS (1961). The serial cultivation of human diploid cell strains. Exp Cell Res.

[9] Hayflick L (1965). THE LIMITED IN VITRO LIFETIME OF HUMAN DIPLOID CELL STRAINS. Exp Cell Res.

[10] O'Brien W, Stenman G, Sager R (1986). Suppression of tumor growth by senescence in virally transformed human fibroblasts. Proc Natl Acad Sci.

[11] Sager R (1991). Senescence as a mode of tumor suppression. Environ Health Perspect.

[12] Collado M, Gil J, Efeyan A, Guerra C, Schuhmacher AJ, Barradas M (2005). Tumour biology: senescence in premalignant tumours. Nature.

[13] Michaloglou C, Vredeveld LC, Soengas MS, Denoyelle C, Kuilman T, van der Horst CM (2005). BRAFE600-associated senescence-like cell cycle arrest of human naevi. Nature.

[14] Choi J, Shendrik I, Peacocke M, Peehl D, Buttyan R, Ikeguchi EF (2000). Expression of senescence-associated beta-galactosidase in enlarged prostates from men with benign prostatic hyperplasia. Urology.

[15] Fujita K, Mondal AM, Horikawa I, Nguyen GH, Kumamoto K, Sohn JJ (2009). p53 isoforms Delta133p53 and p53beta are endogenous regulators of replicative cellular senescence. Nat Cell Biol.

[16] Kuilman T, Michaloglou C, Vredeveld LC, Douma S, van Doorn R, Desmet CJ (2008). Oncogene-induced senescence relayed by an interleukin-dependent inflammatory network. Cell.

[17] Bartkova J, Rezaei N, Liontos M, Karakaidos P, Kletsas D, Issaeva N (2006). Oncogene-induced senescence is part of the tumorigenesis barrier imposed by DNA damage checkpoints. Nature.

[18] Majumder PK, Grisanzio C, O'Connell F, Barry M, Brito JM, Xu Q (2008). A prostatic intraepithelial neoplasia-dependent p27 Kip1 checkpoint induces senescence and inhibits cell proliferation and cancer progression. Cancer Cell.

[19] Serrano M, Lin AW, McCurrach ME, Beach D, Lowe SW (1997). Oncogenic ras provokes premature cell senescence associated with accumulation of p53 and p16INK4a. Cell.

[20] Braig M, Lee S, Loddenkemper C, Rudolph C, Peters AH, Schlegelberger B (2005). Oncogene-induced senescence as an initial barrier in lymphoma development. Nature.

[21] Chen Z, Trotman LC, Shaffer D, Lin HK, Dotan ZA, Niki M (2005). Crucial role of p53-dependent cellular senescence in suppression of Pten-deficient tumorigenesis. Nature.

[22] Goel VK, Ibrahim N, Jiang G, Singhal M, Fee S, Flotte T (2009). Melanocytic nevus-like hyperplasia and melanoma in transgenic BRAFV600E mice. Oncogene.

[23] Dankort D, Filenova E, Collado M, Serrano M, Jones K, McMahon M (2007). A new mouse model to explore the initiation, progression, and therapy of BRAFV600E-induced lung tumors. Genes Dev.

[24] Morton JP, Timpson P, Karim SA, Ridgway RA, Athineos D, Doyle B (2010). Mutant p53 drives metastasis and overcomes growth arrest/senescence in pancreatic cancer. Proc Natl Acad Sci U S A.

[25] Guccini I, Revandkar A, D'Ambrosio M, Colucci M, Pasquini E, Mosole S (2021). Senescence Reprogramming by TIMP1 Deficiency Promotes Prostate Cancer Metastasis. Cancer Cell.

[26] Demaria M, O'Leary MN, Chang J, Shao L, Liu S, Alimirah F (2017). Cellular Senescence Promotes Adverse Effects of Chemotherapy and Cancer Relapse. Cancer Discov.

[27] Coppé JP, Kauser K, Campisi J, Beauséjour CM (2006). Secretion of vascular endothelial growth factor by primary human fibroblasts at senescence. J Biol Chem.

[28] Krtolica A, Parrinello S, Lockett S, Desprez PY, Campisi J (2001). Senescent fibroblasts promote epithelial cell growth and tumorigenesis: a link between cancer and aging. Proc Natl Acad Sci U S A.

[29] Liu D, Hornsby PJ (2007). Senescent human fibroblasts increase the early growth of xenograft tumors via matrix metalloproteinase secretion. Cancer Res.

[30] Faget DV, Ren Q, Stewart SA (2019). Unmasking senescence: context-dependent effects of SASP in cancer. Nat Rev Cancer.

[31] Coppé JP, Desprez PY, Krtolica A, Campisi J (2010). The senescence-associated secretory phenotype: the dark side of tumor suppression. Annu Rev Pathol.

[32] Wang L, Lankhorst L, Bernards R (2022). Exploiting senescence for the treatment of cancer. Nat Rev Cancer.

[33] Galanos P, Vougas K, Walter D, Polyzos A, Maya-Mendoza A, Haagensen EJ (2016). Chronic p53-independent p21 expression causes genomic instability by deregulating replication licensing. Nat Cell Biol.

[34] Milanovic M, Fan DNY, Belenki D, Däbritz JHM, Zhao Z, Yu Y (2018). Senescence-associated reprogramming promotes cancer stemness. Nature.

[35] Patel PL, Suram A, Mirani N, Bischof O, Herbig U (2016). Derepression of hTERT gene expression promotes escape from oncogene-induced cellular senescence. Proc Natl Acad Sci U S A.

[36] Saleh T, Tyutyunyk-Massey L, Gewirtz DA (2019). Tumor Cell Escape from Therapy-Induced Senescence as a Model of Disease Recurrence after Dormancy. Can Res.

[37] Roberson RS, Kussick SJ, Vallieres E, Chen SY, Wu DY (2005). Escape from therapy-induced accelerated cellular senescence in p53-null lung cancer cells and in human lung cancers. Cancer Res.

[38] Campisi J (2005). Senescent cells, tumor suppression, and organismal aging: good citizens, bad neighbors. Cell.

[39] Laconi E, Marongiu F, DeGregori J (2020). Cancer as a disease of old age: changing mutational and microenvironmental landscapes. Br J Cancer.

[40] Chatsirisupachai K, Lesluyes T, Paraoan L, Van Loo P, de Magalhães JP (2021). An integrative analysis of the age-associated multi-omic landscape across cancers. Nat Commun.

[41] Ewald JA, Desotelle JA, Wilding G, Jarrard DF (2010). Therapy-induced senescence in cancer. J Natl Cancer Inst.

[42] Wang B, Kohli J, Demaria M (2020). Senescent Cells in Cancer Therapy: Friends or Foes?. Trends Cancer.

[43] Hanahan D (2022). Hallmarks of Cancer: New Dimensions. Cancer Discov.

[44] Kohli J, Wang B, Brandenburg SM, Basisty N, Evangelou K, Varela-Eirin M (2021). Algorithmic assessment of cellular senescence in experimental and clinical specimens. Nat Protoc.

[45] Evangelou K, Lougiakis N, Rizou SV, Kotsinas A, Kletsas D, Muñoz-Espín D (2017). Robust, universal biomarker assay to detect senescent cells in biological specimens. Aging Cell.

[46] Debacq-Chainiaux F, Erusalimsky JD, Campisi J, Toussaint O (2009). Protocols to detect senescence-associated beta-galactosidase (SA-betagal) activity, a biomarker of senescent cells in culture and in vivo. Nat Protoc.

[47] Robbins E, Levine EM, Eagle H (1970). Morphologic changes accompanying senescence of cultured human diploid cells. J Exp Med.

[48] Dimri GP, Lee X, Basile G, Acosta M, Scott G, Roskelley C (1995). A biomarker that identifies senescent human cells in culture and in aging skin in vivo. Proc Natl Acad Sci U S A.

[49] Hall BM, Balan V, Gleiberman AS, Strom E, Krasnov P, Virtuoso LP (2017). p16(Ink4a) and senescence-associated β-galactosidase can be induced in macrophages as part of a reversible response to physiological stimuli. Aging (Albany NY).

[50] Kopp HG, Hooper AT, Shmelkov SV, Rafii S (2007). Beta-galactosidase staining on bone marrow. The osteoclast pitfall Histol Histopathol.

[51] Georgakopoulou EA, Tsimaratou K, Evangelou K, Fernandez Marcos PJ, Zoumpourlis V, Trougakos IP (2013). Specific lipofuscin staining as a novel biomarker to detect replicative and stress-induced senescence. A method applicable in cryo-preserved and archival tissues. Aging (Albany NY)..

[52] Severino J, Allen RG, Balin S, Balin A, Cristofalo VJ (2000). Is beta-galactosidase staining a marker of senescence in vitro and in vivo?. Exp Cell Res.

[53] Yang NC, Hu ML (2005). The limitations and validities of senescence associated-beta-galactosidase activity as an aging marker for human foreskin fibroblast Hs68 cells. Exp Gerontol.

[54] Lee BY, Han JA, Im JS, Morrone A, Johung K, Goodwin EC (2006). Senescence-associated beta-galactosidase is lysosomal beta-galactosidase. Aging Cell.

[55] Jung T, Bader N, Grune T (2007). Lipofuscin: formation, distribution, and metabolic consequences. Ann N Y Acad Sci.

[56] Hernandez-Segura A, Nehme J, Demaria M (2018). Hallmarks of Cellular Senescence. Trends Cell Biol.

[57] Saif M, Kwanten WJ, Carr JA, Chen IX, Posada JM, Srivastava A (2020). Non-invasive monitoring of chronic liver disease via near-infrared and shortwave-infrared imaging of endogenous lipofuscin. Nature Biomed Engineering.

[58] Alcorta DA, Xiong Y, Phelps D, Hannon G, Beach D, Barrett JC (1996). Involvement of the cyclin-dependent kinase inhibitor p16 (INK4a) in replicative senescence of normal human fibroblasts. Proc Natl Acad Sci U S A.

[59] Beauséjour CM, Krtolica A, Galimi F, Narita M, Lowe SW, Yaswen P (2003). Reversal of human cellular senescence: roles of the p53 and p16 pathways. Embo j.

[60] Karimian A, Ahmadi Y, Yousefi B (2016). Multiple functions of p21 in cell cycle, apoptosis and transcriptional regulation after DNA damage. DNA Repair (Amst).

[61] Hernandez-Segura A, de Jong TV, Melov S, Guryev V, Campisi J, Demaria M (2017). Unmasking Transcriptional Heterogeneity in Senescent Cells. Curr Biol.

[62] Kumari R, Jat P (2021). Mechanisms of Cellular Senescence: Cell Cycle Arrest and Senescence Associated Secretory Phenotype. Front Cell Dev Biol.

[63] Scholzen T, Gerdes J (2000). The Ki-67 protein: from the known and the unknown. J Cell Physiol.

[64] Sen P, Shah PP, Nativio R, Berger SL (2016). Epigenetic Mechanisms of Longevity and Aging. Cell.

[65] Narita M, Nũnez S, Heard E, Narita M, Lin AW, Hearn SA (2003). Rb-mediated heterochromatin formation and silencing of E2F target genes during cellular senescence. Cell.

[66] Aird KM, Zhang R (2013). Detection of senescence-associated heterochromatin foci (SAHF). Methods Mol Biol.

[67] Zhang R, Chen W, Adams PD (2007). Molecular dissection of formation of senescence-associated heterochromatin foci. Mol Cell Biol.

[68] Fagagna FDAD, Reaper PM, Clay-Farrace L, Fiegler H, Carr P, von Zglinicki T (2003). A DNA damage checkpoint response in telomere-initiated senescence. Nature..

[69] Biran A, Zada L, Abou Karam P, Vadai E, Roitman L, Ovadya Y (2017). Quantitative identification of senescent cells in aging and disease. Aging Cell.

[70] Malavolta M, Giacconi R, Piacenza F, Strizzi S, Cardelli M, Bigossi G (2022). Simple Detection of Unstained Live Senescent Cells with Imaging Flow Cytometry. Cells.

[71] Freund A, Laberge RM, Demaria M, Campisi J (2012). Lamin B1 loss is a senescence-associated biomarker. Mol Biol Cell.

[72] Hernandez-Segura A, Rubingh R, Demaria M (2019). Identification of stable senescence-associated reference genes. Aging Cell.

[73] Coppé JP, Patil CK, Rodier F, Sun Y, Muñoz DP, Goldstein J (2008). Senescence-associated secretory phenotypes reveal cell-nonautonomous functions of oncogenic RAS and the p53 tumor suppressor. PLoS Biol.

[74] Basisty N, Kale A, Jeon OH, Kuehnemann C, Payne T, Rao C (2020). A proteomic atlas of senescence-associated secretomes for aging biomarker development. PLoS Biol.

[75] Basisty N, Kale A, Patel S, Campisi J, Schilling B (2020). The power of proteomics to monitor senescence-associated secretory phenotypes and beyond: toward clinical applications. Expert Rev Proteomics.

[76] Jochems F, Thijssen B, De Conti G, Jansen R, Pogacar Z, Groot K (2021). The Cancer SENESCopedia: A delineation of cancer cell senescence. Cell Rep.

[77] Özcan S, Alessio N, Acar MB, Mert E, Omerli F, Peluso G (2016). Unbiased analysis of senescence associated secretory phenotype (SASP) to identify common components following different genotoxic stresses. Aging (Albany NY).

[78] Amor C, Feucht J, Leibold J, Ho YJ, Zhu C, Alonso-Curbelo D (2020). Senolytic CAR T cells reverse senescence-associated pathologies. Nature.

[79] Smith HW, Marshall CJ (2010). Regulation of cell signalling by uPAR. Nat Rev Mol Cell Biol.

[80] Alix-Panabières C, Pantel K (2021). Liquid Biopsy: From Discovery to Clinical Application. Cancer Discov.

[81] Siravegna G, Marsoni S, Siena S, Bardelli A (2017). Integrating liquid biopsies into the management of cancer. Nat Rev Clin Oncol.

[82] Schafer MJ, Zhang X, Kumar A, Atkinson EJ, Zhu Y, Jachim S, et al. The senescence-associated secretome as an indicator of age and medical risk. JCI Insight. 2020;5(12). 10.1172/jci.insight.133668 PMC7406245 32554926

[83] Wollert KC, Kempf T, Wallentin L (2017). Growth Differentiation Factor 15 as a Biomarker in Cardiovascular Disease. Clin Chem.

[84] Roy R, Yang J, Moses MA (2009). Matrix metalloproteinases as novel biomarkers and potential therapeutic targets in human cancer. J Clin Oncol.

[85] Shahim P, Blennow K, Johansson P, Svensson J, Lista S, Hampel H (2017). Cerebrospinal Fluid Stanniocalcin-1 as a Biomarker for Alzheimer's Disease and Other Neurodegenerative Disorders. Neuromolecular Med.

[86] Borghesan M, Fafián-Labora J, Eleftheriadou O, Carpintero-Fernández P, Paez-Ribes M, Vizcay-Barrena G (2019). Small Extracellular Vesicles Are Key Regulators of Non-cell Autonomous Intercellular Communication in Senescence via the Interferon Protein IFITM3. Cell Rep.

[87] Effenberger T, von der Heyde J, Bartsch K, Garbers C, Schulze-Osthoff K, Chalaris A (2014). Senescence-associated release of transmembrane proteins involves proteolytic processing by ADAM17 and microvesicle shedding. Faseb j.

[88] Takasugi M, Okada R, Takahashi A, Virya Chen D, Watanabe S, Hara E (2017). Small extracellular vesicles secreted from senescent cells promote cancer cell proliferation through EphA2. Nat Commun.

[89] Lehmann BD, Paine MS, Brooks AM, McCubrey JA, Renegar RH, Wang R (2008). Senescence-associated exosome release from human prostate cancer cells. Cancer Res.

[90] Kavanagh EL, Lindsay S, Halasz M, Gubbins LC, Weiner-Gorzel K, Guang MHZ (2017). Protein and chemotherapy profiling of extracellular vesicles harvested from therapeutic induced senescent triple negative breast cancer cells. Oncogenesis.

[91] Jeon OH, Wilson DR, Clement CC, Rathod S, Cherry C, Powell B, et al. Senescence cell–associated extracellular vesicles serve as osteoarthritis disease and therapeutic markers. JCI Insight. 2019;4(7). 10.1172/jci.insight.125019 PMC6483636 30944259

[92] Boukouris S, Mathivanan S (2015). Exosomes in bodily fluids are a highly stable resource of disease biomarkers. Proteomics Clin Appl.

[93] Tkach M, Théry C (2016). Communication by Extracellular Vesicles: Where We Are and Where We Need to Go. Cell.

[94] Yáñez-Mó M, Siljander PR, Andreu Z, Zavec AB, Borràs FE, Buzas EI (2015). Biological properties of extracellular vesicles and their physiological functions. J Extracell Vesicles.

[95] Peneder P, Stütz AM, Surdez D, Krumbholz M, Semper S, Chicard M (2021). Multimodal analysis of cell-free DNA whole-genome sequencing for pediatric cancers with low mutational burden. Nat Commun.

[96] Xie W, Kagiampakis I, Pan L, Zhang YW, Murphy L, Tao Y (2018). DNA Methylation Patterns Separate Senescence from Transformation Potential and Indicate Cancer Risk. Cancer Cell.

[97] Levine ME, Leung D, Minteer C, Gonzalez J. A DNA Methylation Fingerprint of Cellular Senescence. bioRxiv. 2019:674580.

[98] Rostami A, Lambie M, Yu CW, Stambolic V, Waldron JN, Bratman SV (2020). Senescence, Necrosis, and Apoptosis Govern Circulating Cell-free DNA Release Kinetics. Cell Rep.

[99] Aizawa K. Studien über Carbohydrasen, I. I. Die fermentative Hydrolyse des p-nitrophenol-β-galactoside. Enzymologia. 1939;6:321–4.

[100] Horwitz JP, Chua J, Curby RJ, Tomson AJ, Da Rooge MA, Fisher BE (1964). Substrates for Cytochemical Demonstration of Enzyme Activity. I. Some Substituted 3-Indolyl-β-D-glycopyranosides1a. J Medicinal Chem..

[101] Rotman B (1961). Measurement of activity of single molecules of beta-D-galactosidase. Proc Natl Acad Sci U S A.

[102] Rotman B, Zderic JA, Edelstein M (1963). Fluorogenic substrates for beta-D-galactosidases and phosphatases derived from flurescein (3,6-dihydroxyfluoran) and its monomethylether. Proc Natl Acad Sci U S A.

[103] Strachan R, Wood J, Hirschmann R (1962). Synthesis and Properties of 4-Methyl-2-oxo-1,2-benzopyran-7-yl β-D-Galactoside (Galactoside of 4-Methylumbelliferone). J Org Chem.

[104] Zhang J, Li C, Dutta C, Fang M, Zhang S, Tiwari A (2017). A novel near-infrared fluorescent probe for sensitive detection of β-galactosidase in living cells. Anal Chim Acta.

[105] Lozano-Torres B, Galiana I, Rovira M, Garrido E, Chaib S, Bernardos A (2017). An OFF-ON Two-Photon Fluorescent Probe for Tracking Cell Senescence in Vivo. J Am Chem Soc.

[106] Wang Y, Liu J, Ma X, Cui C, Deenik PR, Henderson PKP (2019). Real-time imaging of senescence in tumors with DNA damage. Sci Rep.

[107] Lee HW, Heo CH, Sen D, Byun HO, Kwak IH, Yoon G (2014). Ratiometric two-photon fluorescent probe for quantitative detection of β-galactosidase activity in senescent cells. Anal Chem.

[108] Esterly JR, Standen AC, Pearson B (1967). The histochemical demonstration of intestinal beta-D-fucosidase with 5-bromo-4-chloroindole-3-yl-beta-D-fucopyranoside. J Histochem Cytochem.

[109] Rushton AR, Dawson G (1975). Glycosphinoglipid beta-galactosidases of cultured mammalian cells. Characterization of the enzymes from mouse cell line lmtk and human Lesch-Nyhan fibroblasts. Biochim Biophys Acta..

[110] Hildebrand DG, Lehle S, Borst A, Haferkamp S, Essmann F, Schulze-Osthoff K (2013). α-Fucosidase as a novel convenient biomarker for cellular senescence. Cell Cycle.

[111] Agostini A, Mondragón L, Bernardos A, Martínez-Máñez R, Marcos MD, Sancenón F (2012). Targeted cargo delivery in senescent cells using capped mesoporous silica nanoparticles. Angew Chem Int Ed Engl.

[112] Muñoz-Espín D, Rovira M, Galiana I, Giménez C, Lozano-Torres B, Paez-Ribes M, et al. A versatile drug delivery system targeting senescent cells. EMBO Mol Med. 2018;10(9). 10.15252/emmm.201809355 PMC6127887 30012580

[113] Lozano-Torres B, Blandez JF, Galiana I, García-Fernández A, Alfonso M, Marcos MD (2020). Real-Time In Vivo Detection of Cellular Senescence through the Controlled Release of the NIR Fluorescent Dye Nile Blue. Angew Chem Int Ed Engl.

[114] Thapa RK, Nguyen HT, Jeong JH, Kim JR, Choi HG, Yong CS (2017). Progressive slowdown/prevention of cellular senescence by CD9-targeted delivery of rapamycin using lactose-wrapped calcium carbonate nanoparticles. Sci Rep.

[115] Ekpenyong-Akiba AE, Canfarotta F, Abd HB, Poblocka M, Casulleras M, Castilla-Vallmanya L (2019). Detecting and targeting senescent cells using molecularly imprinted nanoparticles. Nanoscale Horizons.

[116] Ou HL, Hoffmann R, González-López C, Doherty GJ, Korkola JE, Muñoz-Espín D (2021). Cellular senescence in cancer: from mechanisms to detection. Mol Oncol.

[117] Krueger MA, Cotton JM, Zhou B, Wolter K, Schwenck J, Kuehn A (2019). Abstract 1146: [18F]FPyGal: A novel ß-galactosidase specific PET tracer for in vivo imaging of tumor senescence. Cancer Research..

[118] Wang L, Lankhorst L, Bernards R. Exploiting senescence for the treatment of cancer.Nat Rev Cancer. 2022;22:340–55. 10.1038/s41568-022-00450-9. 10.1038/s41568-022-00450-9 35241831

[119] Campisi J (2005). Aging, tumor suppression and cancer: high wire-act!. Mech Ageing Dev.

[120] Giacinti C, Giordano A (2006). RB and cell cycle progression. Oncogene.

[121] Prieur A, Besnard E, Babled A, Lemaitre JM (2011). p53 and p16(INK4A) independent induction of senescence by chromatin-dependent alteration of S-phase progression. Nat Commun.

[122] Acosta JC, O'Loghlen A, Banito A, Guijarro MV, Augert A, Raguz S (2008). Chemokine signaling via the CXCR2 receptor reinforces senescence. Cell.

[123] Dietrich N, Bracken AP, Trinh E, Schjerling CK, Koseki H, Rappsilber J (2007). Bypass of senescence by the polycomb group protein CBX8 through direct binding to the INK4A-ARF locus. Embo j.

[124] Pellegrini G, Dellambra E, Paterna P, Golisano O, Traverso CE, Rama P (2004). Telomerase activity is sufficient to bypass replicative senescence in human limbal and conjunctival but not corneal keratinocytes. Eur J Cell Biol.

[125] Abbas T, Dutta A (2009). p21 in cancer: intricate networks and multiple activities. Nat Rev Cancer.

[126] Giatromanolaki A, Kouroupi M, Balaska K, Koukourakis MI (2020). A Novel Lipofuscin-detecting Marker of Senescence Relates With Hypoxia, Dysregulated Autophagy and With Poor Prognosis in Non-small-cell-lung Cancer. In Vivo.

[127] Dosaka-Akita H, Hommura F, Mishina T, Ogura S, Shimizu M, Katoh H (2001). A risk-stratification model of non-small cell lung cancers using cyclin E, Ki-67, and ras p21: different roles of G1 cyclins in cell proliferation and prognosis. Cancer Res.

[128] Komiya T, Hosono Y, Hirashima T, Masuda N, Yasumitsu T, Nakagawa K (1997). p21 expression as a predictor for favorable prognosis in squamous cell carcinoma of the lung. Clin Cancer Res.

[129] Tong J, Sun X, Cheng H, Zhao D, Ma J, Zhen Q (2011). Expression of p16 in non-small cell lung cancer and its prognostic significance: a meta-analysis of published literatures. Lung Cancer.

[130] Sterlacci W, Tzankov A, Veits L, Zelger B, Bihl MP, Foerster A (2011). A comprehensive analysis of p16 expression, gene status, and promoter hypermethylation in surgically resected non-small cell lung carcinomas. J Thorac Oncol.

[131] Sporn JC, Kustatscher G, Hothorn T, Collado M, Serrano M, Muley T (2009). Histone macroH2A isoforms predict the risk of lung cancer recurrence. Oncogene.

[132] Domen A, Deben C, De Pauw I, Hermans C, Lambrechts H, Verswyvel J, Siozopoulou V, Pauwels P, Demaria M, van de Wiel M, Janssens A, Hendriks JMH, Van Schil P, Vermorken JB, Vandamme T, Prenen H, Peeters M, Lardon F, Wouters A. Prognostic implications of cellular senescence in resected non-small cell lung cancer. Transl Lung Cancer Res. 2022;11(8):1526-39. 10.21037/tlcr-22-192. 10.21037/tlcr-22-192 PMC9459607 36090630

[133] Lin W, Wang X, Wang Z, Shao F, Yang Y, Cao Z, et al. Comprehensive Analysis Uncovers Prognostic and Immunogenic Characteristics of Cellular Senescence for Lung Adenocarcinoma. Frontiers Cell Dev Biol. 2021;9. 10.3389/fcell.2021.780461 PMC8636167 34869385

[134] Althubiti M, Lezina L, Carrera S, Jukes-Jones R, Giblett SM, Antonov A (2014). Characterization of novel markers of senescence and their prognostic potential in cancer. Cell Death Dis..

[135] Wang Q, Wu PC, Dong DZ, Ivanova I, Chu E, Zeliadt S (2013). Polyploidy road to therapy-induced cellular senescence and escape. Int J Cancer.

[136] Wang X, Ma L, Pei X, Wang H, Tang X, Pei JF, et al. Comprehensive assessment of cellular senescence in the tumor microenvironment. Brief Bioinform. 2022;23(3). 10.1093/bib/bbac118 PMC9116224 35419596

[137] Sidi R, Pasello G, Opitz I, Soltermann A, Tutic M, Rehrauer H (2011). Induction of senescence markers after neo-adjuvant chemotherapy of malignant pleural mesothelioma and association with clinical outcome: an exploratory analysis. Eur J Cancer.

[138] Pare R, Soon PS, Shah A, Lee CS (2019). Differential expression of senescence tumour markers and its implications on survival outcomes of breast cancer patients. PLoS ONE.

[139] Milde-Langosch K, Bamberger AM, Rieck G, Kelp B, Löning T (2001). Overexpression of the p16 cell cycle inhibitor in breast cancer is associated with a more malignant phenotype. Breast Cancer Res Treat.

[140] Caffo O, Doglioni C, Veronese S, Bonzanini M, Marchetti A, Buttitta F (1996). Prognostic value of p21(WAF1) and p53 expression in breast carcinoma: an immunohistochemical study in 261 patients with long-term follow-up. Clin Cancer Res.

[141] Winters ZE, Hunt NC, Bradburn MJ, Royds JA, Turley H, Harris AL (2001). Subcellular localisation of cyclin B, Cdc2 and p21(WAF1/CIP1) in breast cancer association with prognosis. Eur J Cancer..

[142] Winters ZE, Leek RD, Bradburn MJ, Norbury CJ, Harris AL (2003). Cytoplasmic p21WAF1/CIP1 expression is correlated with HER-2/ neu in breast cancer and is an independent predictor of prognosis. Breast Cancer Res.

[143] Xia W, Chen JS, Zhou X, Sun PR, Lee DF, Liao Y (2004). Phosphorylation/cytoplasmic localization of p21Cip1/WAF1 is associated with HER2/neu overexpression and provides a novel combination predictor for poor prognosis in breast cancer patients. Clin Cancer Res.

[144] Cardoso F, Kyriakides S, Ohno S, Penault-Llorca F, Poortmans P, Rubio IT (2019). Early breast cancer: ESMO Clinical Practice Guidelines for diagnosis, treatment and follow-up. Ann Oncol.

[145] Harbeck N, Kates RE, Look MP, Meijer-Van Gelder ME, Klijn JG, Krüger A (2002). Enhanced benefit from adjuvant chemotherapy in breast cancer patients classified high-risk according to urokinase-type plasminogen activator (uPA) and plasminogen activator inhibitor type 1 (n = 3424). Cancer Res.

[146] Lavigne AC, Castells M, Mermet J, Kocanova S, Dalvai M, Bystricky K (2014). Increased macroH2A1.1 expression correlates with poor survival of triple-negative breast cancer patients. PLoS One..

[147] te Poele RH, Okorokov AL, Jardine L, Cummings J, Joel SP (2002). DNA damage is able to induce senescence in tumor cells in vitro and in vivo. Cancer Res.

[148] Pohl G, Rudas M, Taucher S, Stranzl T, Steger GG, Jakesz R (2003). Expression of Cell Cycle Regulatory Proteins in Breast Carcinomas Before and After Preoperative Chemotherapy. Breast Cancer Res Treat.

[149] Saleh T, Alhesa A, Al-Balas M, Abuelaish O, Mansour A, Awad H, et al. Expression of therapy-induced senescence markers in breast cancer samples upon incomplete response to neoadjuvant chemotherapy. Bioscience Reports. 2021;41(5). 10.1042/BSR20210079 PMC8725197 33948615

[150] Muñoz DP, Yannone SM, Daemen A, Sun Y, Vakar-Lopez F, Kawahara M, et al. Targetable mechanisms driving immunoevasion of persistent senescent cells link chemotherapy-resistant cancer to aging. JCI Insight. 2019;5(14). 10.1172/jci.insight.124716 PMC6675550 31184599

[151] Sirinian C, Peroukidis S, Kriegsmann K, Chaniotis D, Koutras A, Kriegsmann M (2022). Cellular Senescence in Normal Mammary Gland and Breast Cancer. Implications Cancer Ther Genes.

[152] Feng W, Xiao J, Zhang Z, Rosen DG, Brown RE, Liu J (2007). Senescence and apoptosis in carcinogenesis of cervical squamous carcinoma. Mod Pathol.

[153] Zhang Y, Guo L, Xing P, Chen Y, Li F, Zhu W (2014). Increased expression of oncogene-induced senescence markers during cervical squamous cell cancer development. Int J Clin Exp Pathol.

[154] Bae DS, Cho SB, Kim YJ, Whang JD, Song SY, Park CS (2001). Aberrant expression of cyclin D1 is associated with poor prognosis in early stage cervical cancer of the uterus. Gynecol Oncol.

[155] Cheung TH, Lo KW, Yu MM, Yim SF, Poon CS, Chung TK (2001). Aberrant expression of p21(WAF1/CIP1) and p27(KIP1) in cervical carcinoma. Cancer Lett.

[156] Lu X, Toki T, Konishi I, Nikaido T, Fujii S (1998). Expression of p21WAF1/CIP1 in adenocarcinoma of the uterine cervix: a possible immunohistochemical marker of a favorable prognosis. Cancer.

[157] Pakuła M, Mały E, Uruski P, Witucka A, Bogucka M, Jaroszewska N (2020). Deciphering the Molecular Mechanism of Spontaneous Senescence in Primary Epithelial Ovarian Cancer Cells. Cancers.

[158] Anttila MA, Kosma VM, Hongxiu J, Puolakka J, Juhola M, Saarikoski S (1999). p21/WAF1 expression as related to p53, cell proliferation and prognosis in epithelial ovarian cancer. Br J Cancer.

[159] Uruski P, Mikuła-Pietrasik J, Naumowicz E, Kaźmierczak K, Gaiday AN, Królak J (2021). Patient-Specific Variables Determine the Extent of Cellular Senescence Biomarkers in Ovarian Tumors In Vivo. Biomedicines.

[160] Dong Y, Walsh MD, McGuckin MA, Cummings MC, Gabrielli BG, Wright GR (1997). Reduced expression of retinoblastoma gene product (pRB) and high expression of p53 are associated with poor prognosis in ovarian cancer. Int J Cancer.

[161] Yang G, Rosen DG, Zhang Z, Bast RC, Mills GB, Colacino JA (2006). The chemokine growth-regulated oncogene 1 (Gro-1) links RAS signaling to the senescence of stromal fibroblasts and ovarian tumorigenesis. Proc Natl Acad Sci U S A.

[162] Ferrandina G, Stoler A, Fagotti A, Fanfani F, Sacco R, De Pasqua A (2000). p21WAF1/CIP1 protein expression in primary ovarian cancer. Int J Oncol.

[163] Calvo L, Cheng S, Skulimowski M, Clément I, Portelance L, Zhan Y, et al. Cellular senescence is a central response to cytotoxic chemotherapy in high-grade serous ovarian cancer. bioRxiv. 2018:425199.

[164] Xu Q, Ma P, Hu C, Chen L, Xue L, Wang Z (2012). Overexpression of the DEC1 protein induces senescence in vitro and is related to better survival in esophageal squamous cell carcinoma. PLoS ONE.

[165] Güner D, Sturm I, Hemmati P, Hermann S, Hauptmann S, Wurm R (2003). Multigene analysis of Rb pathway and apoptosis control in esophageal squamous cell carcinoma identifies patients with good prognosis. Int J Cancer.

[166] Bai P, Xiao X, Zou J, Cui L, Bui Nguyen TM, Liu J (2012). Expression of p14(ARF), p15(INK4b), p16(INK4a) and skp2 increases during esophageal squamous cell cancer progression. Exp Ther Med.

[167] Sarbia M, Stahl M, Zimmermann K, Wang L, Fink U, zurHausen A (1998). Expression of p21WAF1 predicts outcome of esophageal cancer patients treated by surgery alone or by combined therapy modalities. Clin Cancer Res..

[168] Zhou L, Niu Z, Wang Y, Zheng Y, Zhu Y, Wang C (2022). Senescence as a dictator of patient outcomes and therapeutic efficacies in human gastric cancer. Cell Death Discov.

[169] Aoyagi K, Koufuji K, Yano S, Murakami N, Miyagi M, Koga A (2003). The expression of p53, p21 and TGF beta 1 in gastric carcinoma. Kurume Med J.

[170] Ogawa M, Onoda N, Maeda K, Kato Y, Nakata B, Kang SM (2001). A combination analysis of p53 and p21 in gastric carcinoma as a strong indicator for prognosis. Int J Mol Med.

[171] Bennecke M, Kriegl L, Bajbouj M, Retzlaff K, Robine S, Jung A (2010). Ink4a/Arf and oncogene-induced senescence prevent tumor progression during alternative colorectal tumorigenesis. Cancer Cell.

[172] Kriegl L, Neumann J, Vieth M, Greten FR, Reu S, Jung A (2011). Up and downregulation of p16Ink4a expression in BRAF-mutated polyps/adenomas indicates a senescence barrier in the serrated route to colon cancer. Mod Pathol.

[173] Polyak K, Hamilton SR, Vogelstein B, Kinzler KW (1996). Early alteration of cell-cycle-regulated gene expression in colorectal neoplasia. Am J Pathol.

[174] Kellers F, Fernandez A, Konukiewitz B, Schindeldecker M, Tagscherer KE, Heintz A (2022). Senescence-Associated Molecules and Tumor-Immune-Interactions as Prognostic Biomarkers in Colorectal Cancer. Front Med (Lausanne).

[175] Roxburgh CS, Richards CH, MacDonald AI, Powell AG, McGlynn LM, McMillan DC (2013). The in situ local immune response, tumour senescence and proliferation in colorectal cancer. British Journal of Cancer..

[176] Haugstetter AM, Loddenkemper C, Lenze D, Gröne J, Standfuß C, Petersen I (2010). Cellular senescence predicts treatment outcome in metastasised colorectal cancer. Br J Cancer.

[177] Tato-Costa J, Casimiro S, Pacheco T, Pires R, Fernandes A, Alho I (2016). Therapy-Induced Cellular Senescence Induces Epithelial-to-Mesenchymal Transition and Increases Invasiveness in Rectal Cancer. Clin Colorectal Cancer.

[178] Foersch S, Sperka T, Lindner C, Taut A, Rudolph KL, Breier G (2015). VEGFR2 Signaling Prevents Colorectal Cancer Cell Senescence to Promote Tumorigenesis in Mice With Colitis. Gastroenterology.

[179] Bukholm IK, Nesland JM (2000). Protein expression of p53, p21 (WAF1/CIP1), bcl-2, Bax, cyclin D1 and pRb in human colon carcinomas. Virchows Arch.

[180] Ogino S, Kawasaki T, Kirkner G, Ogawa A, Dorfman I, Loda M (2006). Down-regulation of p21 (CDKN1A/CIP1) is inversely associated with microsatellite instability and CpG island methylator phenotype (CIMP) in colorectal cancer. J Pathol.

[181] Edmonston TB, Cuesta KH, Burkholder S, Barusevicius A, Rose D, Kovatich AJ (2000). Colorectal carcinomas with high microsatellite instability: defining a distinct immunologic and molecular entity with respect to prognostic markers. Hum Pathol.

[182] Zirbes TK, Baldus SE, Moenig SP, Nolden S, Kunze D, Shafizadeh ST (2000). Prognostic impact of p21/waf1/cip1 in colorectal cancer. Int J Cancer.

[183] Mitomi H, Mori A, Kanazawa H, Nishiyama Y, Ihara A, Otani Y (2005). Venous invasion and down-regulation of p21(WAF1/CIP1) are associated with metastasis in colorectal carcinomas. Hepatogastroenterology.

[184] Nicolas AM, Pesic M, Engel E, Ziegler PK, Diefenhardt M, Kennel KB (2022). Inflammatory fibroblasts mediate resistance to neoadjuvant therapy in rectal cancer. Cancer Cell.

[185] Braumüller H, Mauerer B, Berlin C, Plundrich D, Marbach P, Cauchy P, et al. Senescent Tumor Cells in the Peritoneal Carcinomatosis Drive Immunosenescence in the Tumor Microenvironment. Frontiers Immunol. 2022;13. 10.3389/fimmu.2022.908449 PMC9279937 35844581

[186] Guerra C, Collado M, Navas C, Schuhmacher AJ, Hernández-Porras I, Cañamero M (2011). Pancreatitis-induced inflammation contributes to pancreatic cancer by inhibiting oncogene-induced senescence. Cancer Cell.

[187] Biankin AV, Kench JG, Morey AL, Lee CS, Biankin SA, Head DR (2001). Overexpression of p21(WAF1/CIP1) is an early event in the development of pancreatic intraepithelial neoplasia. Cancer Res.

[188] Caldwell ME, DeNicola GM, Martins CP, Jacobetz MA, Maitra A, Hruban RH (2012). Cellular features of senescence during the evolution of human and murine ductal pancreatic cancer. Oncogene.

[189] Rielland M, Cantor DJ, Graveline R, Hajdu C, Mara L, Diaz Bde D (2014). Senescence-associated SIN3B promotes inflammation and pancreatic cancer progression. J Clin Invest.

[190] Yildiz G, Arslan-Ergul A, Bagislar S, Konu O, Yuzugullu H, Gursoy-Yuzugullu O (2013). Genome-wide transcriptional reorganization associated with senescence-to-immortality switch during human hepatocellular carcinogenesis. PLoS ONE.

[191] Wiemann SU, Satyanarayana A, Tsahuridu M, Tillmann HL, Zender L, Klempnauer J (2002). Hepatocyte telomere shortening and senescence are general markers of human liver cirrhosis. Faseb j.

[192] Paradis V, Youssef N, Dargère D, Bâ N, Bonvoust F, Deschatrette J (2001). Replicative senescence in normal liver, chronic hepatitis C, and hepatocellular carcinomas. Hum Pathol.

[193] Wagayama H, Shiraki K, Sugimoto K, Ito T, Fujikawa K, Yamanaka T (2002). High expression of p21WAF1/CIP1 is correlated with human hepatocellular carcinoma in patients with hepatitis C virus-associated chronic liver diseases. Hum Pathol.

[194] Lunz JG, Tsuji H, Nozaki I, Murase N, Demetris AJ (2005). An inhibitor of cyclin-dependent kinase, stress-induced p21Waf-1/Cip-1, mediates hepatocyte mito-inhibition during the evolution of cirrhosis. Hepatology.

[195] Plentz RR, Park YN, Lechel A, Kim H, Nellessen F, Langkopf BH (2007). Telomere shortening and inactivation of cell cycle checkpoints characterize human hepatocarcinogenesis. Hepatology.

[196] Eggert T, Wolter K, Ji J, Ma C, Yevsa T, Klotz S (2016). Distinct Functions of Senescence-Associated Immune Responses in Liver Tumor Surveillance and Tumor Progression. Cancer Cell.

[197] Jiang Y, Luo K, Xu J, Shen X, Gao Y, Fu W, et al. Integrated Analysis Revealing the Senescence-Mediated Immune Heterogeneity of HCC and Construction of a Prognostic Model Based on Senescence-Related Non-Coding RNA Network. Frontiers Oncol. 2022;12. 10.3389/fonc.2022.912537 PMC9279728 35847928

[198] Mo Z, Zheng S, Lv Z, Zhuang Y, Lan X, Wang F (2016). Senescence marker protein 30 (SMP30) serves as a potential prognostic indicator in hepatocellular carcinoma. Sci Rep.

[199] Sasaki M, Nakanuma Y (2015). Cellular senescence in biliary pathology. Special emphasis on expression of a polycomb group protein EZH2 and a senescent marker p16INK4a in bile ductular tumors and lesions. Histol Histopathol..

[200] Yamaguchi J, Sasaki M, Harada K, Zen Y, Sato Y, Ikeda H (2009). Papillary hyperplasia of the gallbladder in pancreaticobiliary maljunction represents a senescence-related lesion induced by lysolecithin. Lab Invest.

[201] Sasaki M, Yamaguchi J, Itatsu K, Ikeda H, Nakanuma Y (2008). Over-expression of polycomb group protein EZH2 relates to decreased expression of p16 INK4a in cholangiocarcinogenesis in hepatolithiasis. J Pathol.

[202] Wagner J, Damaschke N, Yang B, Truong M, Guenther C, McCormick J (2015). Overexpression of the novel senescence marker β-galactosidase (GLB1) in prostate cancer predicts reduced PSA recurrence. PLoS ONE.

[203] Blute ML, Damaschke N, Wagner J, Yang B, Gleave M, Fazli L (2017). Persistence of senescent prostate cancer cells following prolonged neoadjuvant androgen deprivation therapy. PLoS ONE.

[204] Ewald JA, Desotelle JA, Church DR, Yang B, Huang W, Laurila TA (2013). Androgen deprivation induces senescence characteristics in prostate cancer cells in vitro and in vivo. Prostate.

[205] Aaltomaa S, Lipponen P, Eskelinen M, Ala-Opas M, Kosma VM (1999). Prognostic value and expression of p21(waf1/cip1) protein in prostate cancer. Prostate.

[206] Baretton GB, Klenk U, Diebold J, Schmeller N, Löhrs U (1999). Proliferation- and apoptosis-associated factors in advanced prostatic carcinomas before and after androgen deprivation therapy: prognostic significance of p21/WAF1/CIP1 expression. Br J Cancer.

[207] Matsushita K, Cha EK, Matsumoto K, Baba S, Chromecki TF, Fajkovic H (2011). Immunohistochemical biomarkers for bladder cancer prognosis. Int J Urol.

[208] Korkolopoulou P, Konstantinidou AE, Thomas-Tsagli E, Christodoulou P, Kapralos P, Davaris P (2000). WAF1/p21 protein expression is an independent prognostic indicator in superficial and invasive bladder cancer. Appl Immunohistochem Mol Morphol.

[209] Gray-Schopfer VC, Cheong SC, Chong H, Chow J, Moss T, Abdel-Malek ZA (2006). Cellular senescence in naevi and immortalisation in melanoma: a role for p16?. Br J Cancer.

[210] Courtois-Cox S, Genther Williams SM, Reczek EE, Johnson BW, McGillicuddy LT, Johannessen CM (2006). A negative feedback signaling network underlies oncogene-induced senescence. Cancer Cell.

[211] Sparrow LE, Eldon MJ, English DR, Heenan PJ (1998). p16 and p21WAF1 protein expression in melanocytic tumors by immunohistochemistry. Am J Dermatopathol.

[212] Sanki A, Li W, Colman M, Karim RZ, Thompson JF, Scolyer RA (2007). Reduced expression of p16 and p27 is correlated with tumour progression in cutaneous melanoma. Pathology.

[213] Pavey SJ, Cummings MC, Whiteman DC, Castellano M, Walsh MD, Gabrielli BG (2002). Loss of p16 expression is associated with histological features of melanoma invasion. Melanoma Res.

[214] Talve L, Sauroja I, Collan Y, Punnonen K, Ekfors T (1997). Loss of expression of the p16INK4/CDKN2 gene in cutaneous malignant melanoma correlates with tumor cell proliferation and invasive stage. Int J Cancer.

[215] Straume O, Sviland L, Akslen LA (2000). Loss of nuclear p16 protein expression correlates with increased tumor cell proliferation (Ki-67) and poor prognosis in patients with vertical growth phase melanoma. Clin Cancer Res.

[216] Straume O, Akslen LA (1997). Alterations and prognostic significance of p16 and p53 protein expression in subgroups of cutaneous melanoma. Int J Cancer.

[217] Vizioli MG, Possik PA, Tarantino E, Meissl K, Borrello MG, Miranda C (2011). Evidence of oncogene-induced senescence in thyroid carcinogenesis. Endocr Relat Cancer.

[218] Kim YH, Choi YW, Han JH, Lee J, Soh EY, Park SH (2014). TSH signaling overcomes B-RafV600E-induced senescence in papillary thyroid carcinogenesis through regulation of DUSP6. Neoplasia.

[219] Kim YH, Choi YW, Lee J, Soh EY, Kim J-H, Park TJ (2017). Senescent tumor cells lead the collective invasion in thyroid cancer. Nat Commun.

[220] Knösel T, Altendorf-Hofmann A, Lindner L, Issels R, Hermeking H, Schuebbe G (2014). Loss of p16(INK4a) is associated with reduced patient survival in soft tissue tumours, and indicates a senescence barrier. J Clin Pathol.

[221] Lv Y, Wu L, Jian H, Zhang C, Lou Y, Kang Y, et al. Identification and characterization of aging/senescence-induced genes in osteosarcoma and predicting clinical prognosis. Frontiers Immunol. 2022;13. 10.3389/fimmu.2022.997765 PMC9579318 36275664

[222] Macher-Goeppinger S, Bermejo JL, Schirmacher P, Pahernik S, Hohenfellner M, Roth W (2013). Senescence-associated protein p400 is a prognostic marker in renal cell carcinoma. Oncol Rep.

[223] Zhu Y, Xu L, Zhang J, Hu X, Liu Y, Yin H (2013). Sunitinib induces cellular senescence via p53/Dec1 activation in renal cell carcinoma cells. Cancer Sci.

[224] Jacob K, Quang-Khuong D-A, Jones DTW, Witt H, Lambert S, Albrecht S (2011). Genetic Aberrations Leading to MAPK Pathway Activation Mediate Oncogene-Induced Senescence in Sporadic Pilocytic Astrocytomas. Clin Cancer Res.

[225] Buhl JL, Selt F, Hielscher T, Guiho R, Ecker J, Sahm F (2019). The Senescence-associated Secretory Phenotype Mediates Oncogene-induced Senescence in Pediatric Pilocytic Astrocytoma. Clin Cancer Res.

[226] Schiffman JD, Hodgson JG, VandenBerg SR, Flaherty P, Polley M-YC, Yu M (2010). Oncogenic BRAF Mutation with CDKN2A Inactivation Is Characteristic of a Subset of Pediatric Malignant Astrocytomas. Cancer Res..

[227] Bax DA, Mackay A, Little SE, Carvalho D, Viana-Pereira M, Tamber N (2010). A Distinct Spectrum of Copy Number Aberrations in Pediatric High-Grade Gliomas. Clin Cancer Res.

[228] Reinhardt A, Stichel D, Schrimpf D, Sahm F, Korshunov A, Reuss DE (2018). Anaplastic astrocytoma with piloid features, a novel molecular class of IDH wildtype glioma with recurrent MAPK pathway, CDKN2A/B and ATRX alterations. Acta Neuropathol.

[229] Brat DJ, Aldape K, Colman H, Figrarella-Branger D, Fuller GN, Giannini C (2020). cIMPACT-NOW update 5: recommended grading criteria and terminologies for IDH-mutant astrocytomas. Acta Neuropathol.

[230] Reis GF, Pekmezci M, Hansen HM, Rice T, Marshall RE, Molinaro AM (2015). CDKN2A Loss Is Associated With Shortened Overall Survival in Lower-Grade (World Health Organization Grades II–III) Astrocytomas. J Neuropathol Exp Neurol.

[231] Gonzalez-Meljem JM, Haston S, Carreno G, Apps JR, Pozzi S, Stache C (2017). Stem cell senescence drives age-attenuated induction of pituitary tumours in mouse models of paediatric craniopharyngioma. Nat Commun.

[232] Apps JR, Carreno G, Gonzalez-Meljem JM, Haston S, Guiho R, Cooper JE (2018). Tumour compartment transcriptomics demonstrates the activation of inflammatory and odontogenic programmes in human adamantinomatous craniopharyngioma and identifies the MAPK/ERK pathway as a novel therapeutic target. Acta Neuropathol.

[233] Tamayo-Orrego L, Wu CL, Bouchard N, Khedher A, Swikert SM, Remke M (2016). Evasion of Cell Senescence Leads to Medulloblastoma Progression. Cell Rep.

[234] Kool M, Jones DT, Jäger N, Northcott PA, Pugh TJ, Hovestadt V (2014). Genome sequencing of SHH medulloblastoma predicts genotype-related response to smoothened inhibition. Cancer Cell.

[235] Carreno G, Guiho R, Martinez-Barbera JP (2021). Cell senescence in neuropathology: A focus on neurodegeneration and tumours. Neuropathol Appl Neurobiol.

[236] Hilton DA, Penney M, Evans B, Sanders H, Love S (2002). Evaluation of molecular markers in low-grade diffuse astrocytomas: loss of p16 and retinoblastoma protein expression is associated with short survival. Am J Surg Pathol.

[237] Coppola D, Balducci L, Chen DT, Loboda A, Nebozhyn M, Staller A (2014). Senescence-associated-gene signature identifies genes linked to age, prognosis, and progression of human gliomas. J Geriatr Oncol.

[238] Salam R, Saliou A, Bielle F, Bertrand M, Antoniewski C, Carpentier C, et al. Cellular senescence in malignant cells promotes tumor progression in mouse and patient Glioblastoma. bioRxiv. 2022:2022.05.18.492465. 10.1038/s41467-023-36124-9 PMC9883514 36707509

[239] Jung JM, Bruner JM, Ruan S, Langford LA, Kyritsis AP, Kobayashi T (1995). Increased levels of p21WAF1/Cip1 in human brain tumors. Oncogene.

[240] Korkolopoulou P, Kouzelis K, Christodoulou P, Papanikolaou A, Thomas-Tsagli E (1998). Expression of retinoblastoma gene product and p21 (WAF1/Cip 1) protein in gliomas: correlations with proliferation markers, p53 expression and survival. Acta Neuropathol.

[241] Lewis JS, Thorstad WL, Chernock RD, Haughey BH, Yip JH, Zhang Q (2010). p16 positive oropharyngeal squamous cell carcinoma:an entity with a favorable prognosis regardless of tumor HPV status. Am J Surg Pathol.

[242] Machiels JP, René Leemans C, Golusinski W, Grau C, Licitra L, Gregoire V (2020). Squamous cell carcinoma of the oral cavity, larynx, oropharynx and hypopharynx: EHNS-ESMO-ESTRO Clinical Practice Guidelines for diagnosis, treatment and follow-up. Ann Oncol.

[243] Natarajan E, Saeb M, Crum CP, Woo SB, McKee PH, Rheinwald JG (2003). Co-expression of p16(INK4A) and laminin 5 gamma2 by microinvasive and superficial squamous cell carcinomas in vivo and by migrating wound and senescent keratinocytes in culture. Am J Pathol.

[244] Angiero F, Berenzi A, Benetti A, Rossi E, del Sordo R, Sidoni A (2008). Expression of P16, P53 and Ki-67 Proteins in the Progression of Epithelial Dysplasia of the Oral Cavity. Anticancer Res.

[245] Soni S, Kaur J, Kumar A, Chakravarti N, Mathur M, Bahadur S (2005). Alterations of rb pathway components are frequent events in patients with oral epithelial dysplasia and predict clinical outcome in patients with squamous cell carcinoma. Oncology.

[246] Reed AL, Califano J, Cairns P, Westra WH, Jones RM, Koch W (1996). High Frequency of p16 (CDKN2/MTS-1/INK4A) Inactivation in Head and Neck Squamous Cell Carcinoma 1, 2. Can Res.

[247] Schenker H, Büttner-Herold M, Fietkau R, Distel LV (2017). Cell-in-cell structures are more potent predictors of outcome than senescence or apoptosis in head and neck squamous cell carcinomas. Radiat Oncol.

[248] Wang J, Zhou C-C, Sun H-C, Li Q, Hu J-D, Jiang T, et al. Identification of several senescence-associated genes signature in head and neck squamous cell carcinoma. J Clin Lab Anal.n/a(n/a):e24555. 10.1002/jcla.24555 PMC9279997 35692082

[249] Fischer CA, Jung M, Zlobec I, Green E, Storck C, Tornillo L (2011). Co-overexpression of p21 and Ki-67 in head and neck squamous cell carcinoma relative to a significantly poor prognosis. Head Neck.

[250] Zhang M, Li J, Wang L, Tian Z, Zhang P, Xu Q (2013). Prognostic significance of p21, p27 and survivin protein expression in patients with oral squamous cell carcinoma. Oncol Lett.

[251] Hafkamp HC, Mooren JJ, Claessen SM, Klingenberg B, Voogd AC, Bot FJ (2009). P21 Cip1/WAF1 expression is strongly associated with HPV-positive tonsillar carcinoma and a favorable prognosis. Mod Pathol.

[252] Kapranos N, Stathopoulos GP, Manolopoulos L, Kokka E, Papadimitriou C, Bibas A (2001). p53, p21 and p27 protein expression in head and neck cancer and their prognostic value. Anticancer Res.

[253] Goulart-Filho JAV, Montalli VAM, Passador-Santos F, de Araújo NS, de Araújo VC (2019). Role of apoptotic, autophagic and senescence pathways in minor salivary gland adenoid cystic carcinoma. Diagn Pathol.

[254] Schoetz U, Klein D, Hess J, Shnayien S, Spoerl S, Orth M (2021). Early senescence and production of senescence-associated cytokines are major determinants of radioresistance in head-and-neck squamous cell carcinoma. Cell Death Dis.

[255] Rao SG, Jackson JG (2016). SASP: Tumor Suppressor or Promoter? Yes!. Trends Cancer.

[256] Chien Y, Scuoppo C, Wang X, Fang X, Balgley B, Bolden JE (2011). Control of the senescence-associated secretory phenotype by NF-kappaB promotes senescence and enhances chemosensitivity. Genes Dev.

[257] Herranz N, Gallage S, Mellone M, Wuestefeld T, Klotz S, Hanley CJ (2015). mTOR regulates MAPKAPK2 translation to control the senescence-associated secretory phenotype. Nat Cell Biol.

[258] Laberge RM, Sun Y, Orjalo AV, Patil CK, Freund A, Zhou L (2015). MTOR regulates the pro-tumorigenic senescence-associated secretory phenotype by promoting IL1A translation. Nat Cell Biol.

[259] Freund A, Patil CK, Campisi J (2011). p38MAPK is a novel DNA damage response-independent regulator of the senescence-associated secretory phenotype. Embo j.

[260] Zacarias-Fluck MF, Morancho B, Vicario R, Luque Garcia A, Escorihuela M, Villanueva J, et al. Effect of Cellular Senescence on the Growth of HER2-Positive Breast Cancers. J Natl Cancer Inst. 2015;107(5). 10.1093/jnci/djv020 25972601

[261] Orjalo AV, Bhaumik D, Gengler BK, Scott GK, Campisi J (2009). Cell surface-bound IL-1alpha is an upstream regulator of the senescence-associated IL-6/IL-8 cytokine network. Proc Natl Acad Sci U S A.

[262] Acosta JC, Banito A, Wuestefeld T, Georgilis A, Janich P, Morton JP (2013). A complex secretory program orchestrated by the inflammasome controls paracrine senescence. Nat Cell Biol.

[263] Ruhland MK, Loza AJ, Capietto AH, Luo X, Knolhoff BL, Flanagan KC, Belt BA, Alspach E, Leahy K, Luo J, Schaffer A, Edwards JR, Longmore G, Faccio R, DeNardo DG, Stewart SA. Stromal senescence establishes an immunosuppressive microenvironment that drives tumorigenesis. Nat Commun. 2016;7:11762. 10.1038/ncomms11762. 10.1038/ncomms11762 PMC4899869 27272654

[264] Ruhland MK, Loza AJ, Capietto AH, Luo X, Knolhoff BL, Flanagan KC (2016). Stromal senescence establishes an immunosuppressive microenvironment that drives tumorigenesis. Nat Commun.

[265] Di Mitri D, Toso A, Chen JJ, Sarti M, Pinton S, Jost TR (2014). Tumour-infiltrating Gr-1+ myeloid cells antagonize senescence in cancer. Nature.

[266] Jackson JG, Pant V, Li Q, Chang LL, Quintás-Cardama A, Garza D (2012). p53-mediated senescence impairs the apoptotic response to chemotherapy and clinical outcome in breast cancer. Cancer Cell.

[267] Bertheau P, Plassa F, Espié M, Turpin E, de Roquancourt A, Marty M (2002). Effect of mutated TP53 on response of advanced breast cancers to high-dose chemotherapy. Lancet.

[268] Bertheau P, Turpin E, Rickman DS, Espié M, de Reyniès A, Feugeas JP (2007). Exquisite sensitivity of TP53 mutant and basal breast cancers to a dose-dense epirubicin-cyclophosphamide regimen. PLoS Med.

[269] Zhou D, Borsa M, Simon AK (2021). Hallmarks and detection techniques of cellular senescence and cellular ageing in immune cells. Aging Cell.

[270] Gonzalez-Meljem JM, Apps JR, Fraser HC, Martinez-Barbera JP (2018). Paracrine roles of cellular senescence in promoting tumourigenesis. Br J Cancer.

[271] Lee S, Schmitt CA (2019). The dynamic nature of senescence in cancer. Nat Cell Biol.

[272] Niedernhofer LJ, Robbins PD (2018). Senotherapeutics for healthy ageing. Nature Rev Drug Discov..

[273] Cui M, Zhang DY (2021). Artificial intelligence and computational pathology. Lab Invest.

[274] Domen A, Quatannens D, Zanivan S, Deben C, Van Audenaerde J, Smits E, et al. Cancer-Associated Fibroblasts as a Common Orchestrator of Therapy Resistance in Lung and Pancreatic Cancer. Cancers (Basel). 2021;13(5). 10.3390/cancers13050987 PMC7956441 33673405

[275] Drost J, Clevers H (2018). Organoids in cancer research. Nat Rev Cancer.

[276] Moncada R, Barkley D, Wagner F, Chiodin M, Devlin JC, Baron M (2020). Integrating microarray-based spatial transcriptomics and single-cell RNA-seq reveals tissue architecture in pancreatic ductal adenocarcinomas. Nat Biotechnol.

